# An Innovative Differentiated Creative Search Based on Collaborative Development and Population Evaluation

**DOI:** 10.3390/biomimetics10050260

**Published:** 2025-04-23

**Authors:** Xinyu Cai, Chaoyong Zhang

**Affiliations:** 1 College of Business, Jiaxing University, Jiaxing 314001, China; caixinyu@zjxu.edu.cn; 2 School of Civil Engineering and Architecture, Jiaxing Nanhu University, Jiaxing 314000, China

**Keywords:** differentiated creative search, metaheuristic algorithm, engineering optimization problems, collaborative development mechanism, linear population size reduction

## Abstract

In real-world applications, many complex problems can be formulated as mathematical optimization challenges, and efficiently solving these problems is critical. Metaheuristic algorithms have proven highly effective in addressing a wide range of engineering issues. The differentiated creative search is a recently proposed evolution-based meta-heuristic algorithm with certain advantages. However, it also has limitations, including weakened population diversity, reduced search efficiency, and hindrance of comprehensive exploration of the solution space. To address the shortcomings of the DCS algorithm, this paper proposes a multi-strategy differentiated creative search (MSDCS) based on the collaborative development mechanism and population evaluation strategy. First, this paper proposes a collaborative development mechanism that organically integrates the estimation distribution algorithm and DCS to compensate for the shortcomings of the DCS algorithm’s insufficient exploration ability and its tendency to fall into local optimums through the guiding effect of dominant populations, and to improve the quality of the DCS algorithm’s search efficiency and solution at the same time. Secondly, a new population evaluation strategy is proposed to realize the coordinated transition between exploitation and exploration through the comprehensive evaluation of fitness and distance. Finally, a linear population size reduction strategy is incorporated into DCS, which significantly improves the overall performance of the algorithm by maintaining a large population size at the initial stage to enhance the exploration capability and extensive search of the solution space, and then gradually decreasing the population size at the later stage to enhance the exploitation capability. A series of validations was conducted on the CEC2018 test set, and the experimental results were analyzed using the Friedman test and Wilcoxon rank sum test. The results show the superior performance of MSDCS in terms of convergence speed, stability, and global optimization. In addition, MSDCS is successfully applied to several engineering constrained optimization problems. In all cases, MSDCS outperforms the basic DCS algorithm with fast convergence and strong robustness, emphasizing its superior efficacy in practical applications.

## 1. Introduction

With the rapid advancement of society, academic research and engineering applications are increasingly confronted with complex optimization problems [1,2]. Solving an optimization problem is the process of determining the optimal solution from among many feasible solutions in a given problem space [3]. Optimization problems are centered on maximizing or minimizing one or more dimensional objective functions while satisfying a set of constraints. Such problems are widespread in fields such as engineering, medicine, economics, and agriculture [4]. However, real-world optimization problems are often extremely complicated due to complex properties such as multivariate, multi-objective, multi-constraint, non-linear, multi-peaked, and non-differentiable [5]. Mathematically, many optimization problems are classified as NP-hard, which implies that the exact solution is computationally resource-intensive and usually cannot be achieved in polynomial time [6,7]. Optimization methods are usually divided into two main categories: deterministic and stochastic algorithms. Deterministic algorithms determine the globally optimal solution using rigorous mathematical techniques such as linear programming, nonlinear programming, branch-and-bound, and dynamic programming [8]. They always produce the same output for a given input and rigorously search for an exact solution to the problem. Although such algorithms often produce optimal solutions, they are computationally demanding, especially when applied to large datasets, and often require longer execution times. In addition, they rely on ideal problem conditions such as linearity, convexity, and differentiability [9]. When dealing with non-differentiable objective functions or problems with multiple local optima, deterministic algorithms tend to fall into local optimality and thus exhibit limited adaptability [10]. Given the increasingly complex and diverse problem space, there is an urgent need for more efficient, stable, and highly portable algorithms to overcome these challenges. Meta-heuristic algorithms are stochastic algorithms. Unlike traditional optimization methods, metaheuristic algorithms require no precise mathematical properties, such as gradient information. Instead, they utilize probabilistic strategies to iteratively explore and exploit the problem space, incrementally improving the quality of the solution. By balancing global exploration (extensive search in the solution space) with local exploitation (fine-tuning of promising solutions), these algorithms effectively mitigate the risk of premature convergence to suboptimal solutions [11]. In addition, the non-derivative or non-gradient character of metaheuristic algorithms allows these algorithms to be quickly applied to optimization problems in a variety of complex scenarios without paying attention to the specific structure of the problem [12]. They can obtain near-optimal solutions while reducing computational resource requirements, making them suitable for complex real-world optimization problems such as path planning [13,14], wireless sensor network coverage [15,16], image segmentation [17,18], and feature selection [19,20]. By drawing inspiration from nature and social systems, metaheuristic algorithms provide diverse and flexible solutions to complex optimization problems.

**Figure 1:**
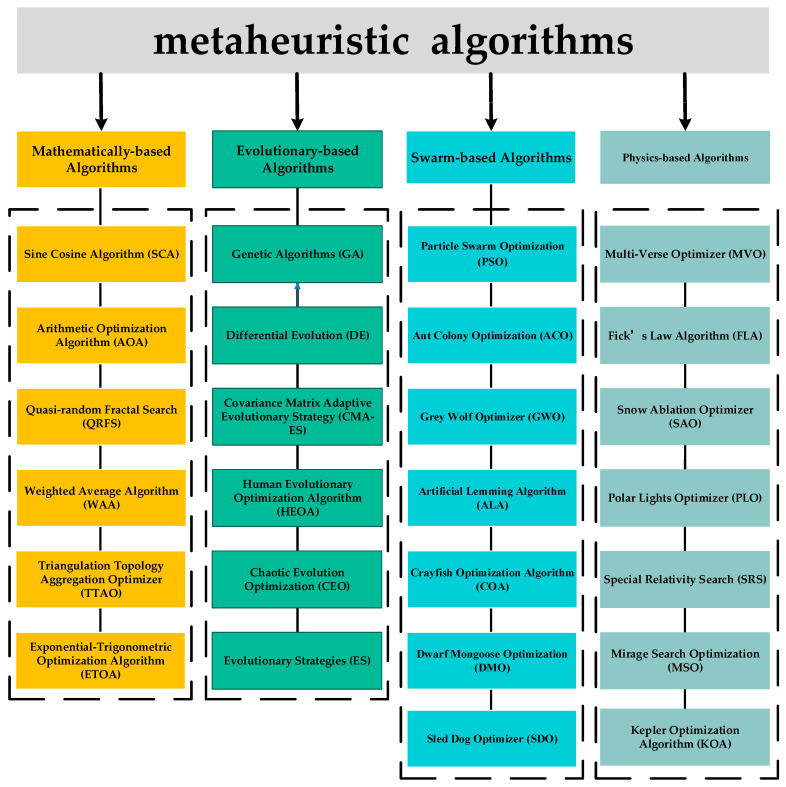
Classification of metaheuristic algorithm.

Metaheuristic algorithms represent a class of stochastic optimization techniques inspired by natural phenomena, physical laws, or human behavior. Metaheuristic algorithms can be categorized into four primary groups based on their inspiration (as shown in Figure 1). Evolution-based algorithms: These algorithms are inspired by the principles of natural evolution, including selection, mutation, and crossover. These algorithms mimic the process of natural selection, where the fittest individuals are selected to create the next generation. Examples include the Genetic Algorithm (GA) [21], Evolutionary Strategy (ES) [22], Differential Evolution (DE) [23], Covariance Matrix Adaptive Evolutionary Strategy (CMA-ES) [24], Human Evolutionary Optimization Algorithm (HEOA) [25], and Chaotic Evolution Optimization (CEO) [26]. Physics-based algorithms: these draw on the laws of physics or chemical reaction mechanisms, including Multi-Verse Optimizer (MVO) [27], Fick’s Law Algorithm (FLA) [28], Snow Ablation Optimization (SAO) [29], Polar Lights Optimizer (PLO) [30], Special Relativity Search (SRS) [31], Mirage Search Optimization (MSO) [32], and Kepler Optimization Algorithm (KOA) [33]. The third category comprises mathematical-based algorithms, which are inspired by mathematical theories, functions, and formulas, which have demonstrated significant promise in enhancing the computing efficacy of optimization approaches. One of the noteworthy methods in this category is the Sine Cosine Algorithm (SCA) [34], which applies the concept of trigonometric functions to create an algorithmic model. Arithmetic Optimization Algorithm (AOA) [35], Quasi-random Fractal Search (QRFS) [36], Weighted Average Algorithm (WAA) [37], Triangulation Topology Aggregation Optimizer (TTAO) [38], and Exponential-Trigonometric Optimization Algorithm (ETOA) [39] are other instances in this category. Swarm-based algorithms focus on group collaboration and information exchange to find global optima, simulating the collective social behavior of foraging, reproduction, and avoidance of natural enemies in groups of organisms. Particle Swarm Optimization (PSO) [40] is one of the classical instances in which the foraging behavioral patterns of bird flocks are simulated to locate the ideal solution. Other prominent algorithms in this category comprise Ant Colony Optimization (ACO) [41], Grey Wolf Optimizer (GWO) [42], Artificial Lemming Algorithm (ALA) [43], Crayfish Optimization Algorithm (COA) [44], Dwarf Mongoose Optimization (DMO) [45], Sled Dog Optimizer (SDO) [46], Slime Mould Algorithm (SMA) [47], Tuna Swarm Optimization (TSO) [48], Genghis Khan Shark Optimizer (GKSO) [49], Prairie Dog Optimization Algorithm (PDOA) [50], and Secretary Bird Optimization Algorithm (SBOA) [51].

Their widespread success across various domains reinforces their standing as a superior alternative to traditional optimization methods. Despite significant progress in various areas, metaheuristic algorithms have encountered challenges and problems. First, metaheuristic algorithms can obtain better solutions, but it is difficult to obtain optimal solutions. Second, metaheuristic algorithms are often difficult to balance between exploitation and exploration, which is related to the adaptive nature of their parameters or the focus of the search strategy. The population will homogenize to a single position at some moment, which causes the algorithm to easily fall into a local optimum. Notably, the no free lunch theorem clearly states that no single algorithm can outperform all other algorithms on all possible optimization problems [52]. In other words, optimization algorithms may perform well on some problems but poorly on others. This forces researchers to design or improve efficient algorithms for specific optimization problems with the aim of providing superior solutions.

Differentiated Creative Search (DCS) is an evolution-based meta-heuristic algorithm proposed by Duankhan et al. in 2024 [53]. DCS utilizes differential knowledge acquisition and creative realism to model the algorithm and proposes two different search strategies, divergent and convergent thinking, to implement the problem solution. Although DCS has shown great potential as a metaheuristic algorithm, limitations such as insufficient population diversity, unbalanced exploitation and exploration, and weak global search capability hinder its performance in complex optimization scenarios. To address these challenges, several researchers have systematically improved the algorithm. Chermite et al. designed a hybrid DCS algorithm that utilizes the Newton Raphson method to further improve the quality of the solution and achieve parameter extraction for PV models [54]. Zhang et al. adopted a guided learning strategy to balance the exploitation and exploration of DCS and applied the improved DCS algorithm to optimize the support vector machine model parameters [55]. Liu et al. enhanced the convergence accuracy and the ability to get rid of local optimums by integrating multiple improvement strategies in DCS and solved the agricultural UAV path planning problem [56].

The above studies have enhanced the efficiency of DCS through different methods. However, the inability of DCS to face complex optimization problems is something that exists objectively. Meanwhile, the theory of no free lunch states that a great algorithm may perform well in solving a specific type of optimization problem but may be ineffective for other types of problems. Considering the above factors, this paper proposes a new variant of DCS called Multi-Strategy Differentiated Creative Search (MSDCS). MSDCS combines three improvement techniques to realize a leap in performance. First, this paper proposes a collaborative development mechanism to organically integrate the estimation distribution algorithm and DCS. The defects of the DCS algorithm, including its insufficient exploration ability and the fact that it is easy for it to fall into local optimization, are compensated for by the guiding effect of advantageous populations. At the same time, it improves the search efficiency and the quality of the DCS algorithm. Secondly, a new population evaluation strategy is proposed to realize the coordinated transition between exploitation and exploration through the comprehensive evaluation of fitness and distance. Finally, a linear population size reduction strategy is integrated into DCS, which enhances the exploration capability and searches the solution space extensively by maintaining a large population size at the initial stage. At a later stage, the population size is gradually reduced to enhance the exploitation capability and improve the accuracy and quality of the solution. Meanwhile, the search process is optimized to significantly improve the overall performance of the algorithm. The proposed MSDCS is comprehensively examined on the CEC2018 benchmark test functions and thoroughly compared with different classes of basic and improved algorithms. Statistical tests, including Friedman’s test and the Wilcoxon rank sum test, are performed to analyze the differences between MSDCS and other compared algorithms. Engineering constrained optimization problems confirm the ability of MSDCS to solve realistic optimization problems. The contributions of this paper are as follows:

(1) Proposing a DCS variant, MSDCS, that combines three improved techniques.
(2) Comprehensively examining the performance of MSDCS using the CEC2018 test suite and confirming its superiority through the Friedman test and the Wilcoxon value sum test.
(3) Applying MSDCS successfully to the wireless sensor network coverage problem and engineering constrained optimization problems, showing its adaptability in facing real-world problems.

The structure of this article is as follows: Section 2 delves into the architecture and specifics of the DCS algorithm. The third chapter is the construction of the theoretical framework, which proposes three improvement strategies, including a collaborative development mechanism, a population evaluation strategy, and a linear population size reduction strategy. Meanwhile, Section 3 provides an in-depth analysis of the algorithmic complexity of MSDCS, including both time complexity and space complexity. Section 4 presents ablation experiments on MSDCS, validating the soundness of its design by testing it on CEC2018 test functions. Furthermore, Section 4 employs a comprehensive and rigorous evaluation of the MSDCS algorithm alongside nine competitive algorithms, utilizing the 10-dimensional, 30-dimensional, 50-dimensional, and 100-dimensional CEC-2017 benchmark functions, with a detailed analysis of the experimental results. Section 5 applies the algorithm to engineering-constrained optimization problems. Finally, Section 6 summarizes the entire work, analyzes the potential limitations of the MSDCS algorithm, and outlines future research directions.

## 2. Differentiated Creative Search

DCS starts the search from a set of randomly generated solution sets and deepens the search through three different update strategies as well as differentiated knowledge acquisition strategies. When the stopping criteria are satisfied, DCS ends the search and outputs the optimal solution. The specific structure of DCS is represented as follows.

### 2.1. Initialization

The original DCS algorithm begins by generating a random population with a uniform distribution to initiate the optimization process. The initial population can be determined using the following equation.

(1) $$X_{i,j}=Lb_{j}+\left(Ub_{j}-Lb_{j}\right)\times U,i=1,2,\ldots ,N;j=1,2,\ldots ,D$$

where $X_{i,j}$ denotes the $j^{th}$ dimensional position of the $i^{th}$ individual; U is a randomly generated number within the range [0, 1]. $Ub_{j}$ and $Lb_{j}$ represent the minimum and maximum bounds of the problem, respectively. N is the total number of whole individuals. Unlike other metaheuristic algorithms, DCS evaluates all individuals after initialization and ranks them based on their fitness. In this paper, we take the minimization problem as an example, where individuals with smaller fitness are ranked higher.

### 2.2. Differentiated Knowledge Acquisition

In the differentiated knowledge-acquisition phase, DCS will calculate the knowledge acquisition rate $\eta_{i}^{t}$ at the $t^{th}$ iteration for each individual. The $\eta_{i}^{t}$ affects the knowledge uptake of each individual in different dimensions, represented as follows.

(2) $$\eta_{i}^{t}=\frac{1}{2}\left(round\left(U\times \vartheta_{i}^{t}\right)+\left\{\begin{array}{c} 1,if\;U<\vartheta_{i}^{t}\\ 0,if\;U\geq \vartheta_{i}^{t} \end{array}\right.\right)$$

where $round\left(\cdot \right)$ denotes rounding the given value to the nearest integer. $\vartheta_{i}^{t}$ is a coefficient that describes an individual’s knowledge deficit and can be represented as follows, with the higher-ranked individual having a smaller $\vartheta_{i}^{t}$.

(3) $$\vartheta_{i}^{t}=0.25+0.55\times \sqrt{R_{i}^{t}/N}$$

where $R_{i}^{t}$ is ranking of the $i^{th}$ individual at the $t^{th}$ iteration. After obtaining the knowledge acquisition rate of each individual, the following equation calculates whether each individual learns the corresponding dimension or not.

(4) $$Xnew_{i,j}=\left\{\begin{array}{l} v_{i,j},if\;U<\eta_{i}^{t}∥\;j=j_{rand}\\ X_{i,j},otherwise \end{array}\right.$$

(5) $$j_{rand}=randint\left(1,D\right)$$

where $v_{i,j}$ is the $j^{th}$ dimensional position of the trial vector for each individual after updating at the $t^{th}$ iteration. $Xnew_{i,j}$ is the child individual of each individual after absorbing the knowledge. $j_{rand}$ is an integer randomly chosen from 1 to D and generated once for each iteration.

### 2.3. Creative Realism

DCS divides the population into three segments: a high-level group with good fitness, a medium level general group with intermediate fitness, and the least fit individual. The high-level group contains the top-ranked $N_{s}$ individuals. Creative realism consists of divergent thinking for the high-level group and convergent thinking for the medium-level group. For the normal group, DCS obtains the trial vector v based on the optimal individual by drawing on the knowledge of two different individuals, denoted as follows.

(6) $$v_{i,j}=X_{best,j}+\lambda \times \left(X_{r2,j}-X_{i,j}\right)+\omega \times \left(X_{r1,j}-X_{i,j}\right),i=N_{s}+1,N_{s}+2,\ldots ,N$$

(7) $$\lambda =0.1+0.518\times \left(1-\sqrt{FEs/FEs_{\max}}\right)$$

where $X_{best,j}$ is the $j^{th}$ dimensional position of the best individual in the population. $X_{r1,j}$ is the $j^{th}$ dimensional position of a randomly selected individual in the population that is different from $X_{best}$, $X_{r2}$, and $X_{i}$. $X_{r2,j}$ is the $j^{th}$ dimensional position of a randomly selected individual from the medium level population and $X_{r2}\neq X_{r1}\neq X_{best}\neq X_{i}$. ω is a randomly generated number within the range [0, 1]. $FEs_{\max}$ and FEs denote the maximum number of function evaluations and the current number of function evaluations, respectively. For high-level groups, DCS generates the experiment vectors using an updating method based on the Linnik distribution, denoted as follows.

(8) $$v_{i,j}=X_{r3,j}+Lk\left(\alpha ,\beta \right),i=1,2,\ldots ,N_{s}$$

where $Lk\left(\alpha ,\beta \right)$ symbolizes the Linnik distribution random number generator with control parameters α and β. $X_{r3,j}$ is the $j^{th}$ dimensional position of a randomly selected individual from the whole population and $X_{r3}\neq X_{best}\neq X_{i}$. For the worst individual, DCS generates the trial vector $v_{N}$ using the following equation.

(9) $$v_{N}=Lb+\left(Ub-Lb\right)\times rand\left(1,D\right),if\;rand<p_{c}$$

where $v_{N}$ denotes the trial vector of the $N^{th}$ individual, which is also the trial vector of the worst individual. $p_{c}$ is a constant with a value of 0.5.

### 2.4. Retrospective Assessment

After generating the trial vectors and learning based on the knowledge acquisition rate, the DCS evaluates the learned individuals and selects them according to the following equation.

(10) $$X_{i}^{t+1}=\left\{\begin{array}{l} Xnew,if\;F\left(Xnew\right)\leq F\left(X_{i}^{t}\right)\\ X_{i}^{t},otherwise \end{array}\right.$$

where $F\left(Xnew\right)$ and $F\left(X_{i}^{t}\right)$ are fitness values of Xnew and $X_{i}^{t}$, respectively.

## 3. Proposed Multi-Strategy Differentiated Creative Search

The DCS algorithm suffers from some shortcomings, limiting its search efficiency. The effective information of the dominant group is ignored throughout the search process, which leads to the evolutionary direction of the population being disordered. In addition, the grouping of DCS is based on the magnitude of fitness, which ignores the factor of distance between different individuals. Fixed population size cannot expand the search in the early stage and narrow the range in the later stage, which weakens the performance of DCS. To overcome these limitations, this paper proposes a multi-strategy DCS algorithm that incorporates three improvement strategies. In this section, the three improvement strategies are described in detail sequentially, accompanied by pseudo-code and a flow chart. The time complexity and space complexity of MSDCS are also demonstrated in this section. 

### 3.1. Population Evaluation Strategy (PES)

The DCS divides the population into three segments based on the fitness ordering and uses this to calculate the knowledge acquisition rate of different individuals. However, DCS relies only on fitness for grouping and ignores the distance information between individuals, making it difficult to maintain population diversity. In order to enrich the population diversity and promote the wide distribution of populations, this paper proposes a population evaluation strategy (PES). PES can consider both fitness and distance to divide populations, and the detailed steps are shown below.

Step 1: Calculate the Euclidean distance between each individual and the optimal individual as in Equation (11).

(11) $$Dis_{i}=\sqrt{{\left(X_{i,1}-X_{best,1}\right)}^{2}+{\left(X_{i,2}-X_{best,2}\right)}^{2}+\ldots +{\left(X_{i,j}-X_{best,j}\right)}^{2}}$$

Step 2: Calculate the score for each individual based on normalized distance and normalized fitness as in Equation (12).

(12) $$Score_{i}=\sigma_{1}\times Norm\left(Dis_{i}\right)+\left(1-\sigma_{1}\right)\times Norm\left(F\left(X_{i}\right)\right)$$

Step 3: Sort each individual based on their score (minimization problem is descending). The coefficient $\sigma_{1}$ determines the extent to which fitness and distance affect the score. In this work, $\sigma_{1}$ is calculated by Equation (13).

(13) $$\sigma_{1}=\mathrm{mod}\left(\frac{FEs_{\max}}{10},FEs\right)/FEs_{\max}\times \left(1-\gamma \right)+\gamma$$

### 3.2. Linear Population Size Reduction Strategy (LPSR)

The size of the population affects the performance of an algorithm. At the initial stage of the algorithm, a larger population size contributes to the population diversity and enhances the exploration capability, enabling the algorithm to search the solution space more extensively and find potential high-quality solution regions. With the gradual reduction of the population size, the exploitation ability of the algorithm is enhanced, and it can focus more on the promising regions to improve the accuracy and quality of the solution, thus effectively balancing the exploration and exploitation ability and making the algorithm converge to the global optimal solution more efficiently and stably. The linear population size reduction strategy (LPSR) is a successful population size adjustment strategy that has been applied in algorithms such as LSHADE. In order to improve the performance of the DCS algorithm, this paper integrates the LPSR strategy into the DCS algorithm. The LPSR strategy is described as follows.

(14) $$N_{new}=round\left(N_{ini}-\frac{FEs}{FEs_{\max}}\times \left(N_{ini}-N_{min}\right)\right)$$

where $N_{ini}$ is the initial population size and $N_{min}$ is the final population size. When the new population size $N_{new}$ is smaller than the current population size N, the population is sorted according to the value of the objective function and the worst $\left(N-N_{new}\right)$ individuals are discarded.

### 3.3. Collaborative Development Mechanism (CDM)

**Figure 2:**
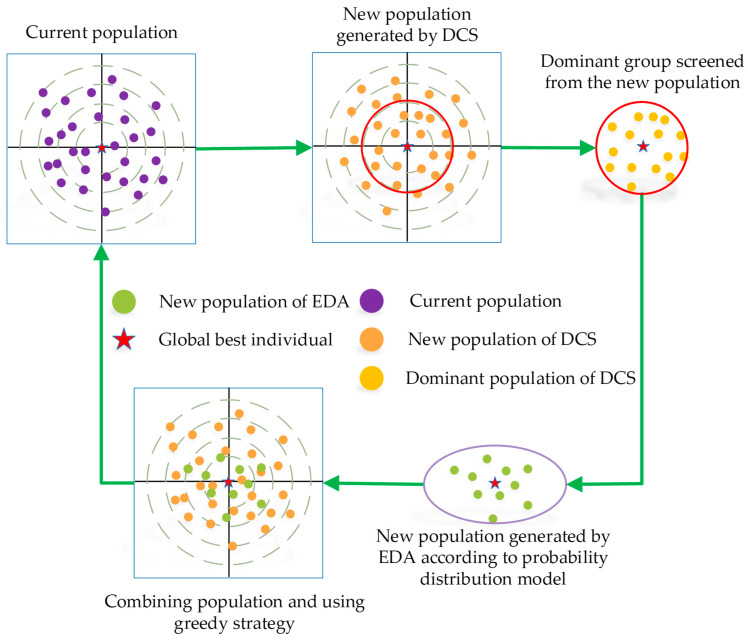
Sketch for collaborative development mechanism.

DCS ignores the guiding effect of the dominant population throughout the search process, which causes the algorithm to fall into local optima more easily, making it difficult to provide high-quality solutions. In order to enhance the global search capability of the DCS algorithm, this paper incorporates the estimation distribution algorithm into DCS. Since the estimated distribution algorithm depends not on an individual but updates the population by virtue of the information of the dominant group, this enables the EDA to have a powerful exploration capability, which is lacking in the DCS algorithm. EDA algorithms can be divided into three phases: selection phase, modeling phase, and generation phase. In the selection phase, EDA ranks the individuals according to the size of their fitness and selects the top fifty percent to form the dominant group $P_{d}$. Then, a Gaussian probability distribution model based on the dominant group is established. Finally, new individuals are generated based on the Gaussian probability distribution model. The mean μ and the covariance matrix C of the Gaussian probability distribution model are calculated as Equations (15) and (16). Equation (17) represents the approach to the new individual generated by the EDA

(15) $$\mu =\frac{1}{\left|P_{d}\right|}\times \sum_{i=1}^{\left|P_{d}\right|}X_{i},X_{i}\in P_{d}$$

(16) $$C=\frac{1}{\left|P_{d}\right|}\times \sum_{i=1}^{\left|P_{d}\right|}\left(X_{i}-\mu \right)\times {\left(X_{i}-\mu \right)}^{T},X_{i}\in P_{d}$$

(17) $$Xnew_{i}=\mu +g,g∼G\left(0,C\right)$$

where $\left|P_{d}\right|$ is the number of dominant groups. Common integration methods include two categories. One category is to replace the original search strategy with a new one. The other category is where the two methods are randomly selected according to probability. These two types of integration methods make it difficult to utilize the existing search strategies at the same time. For this reason, this paper proposes a collaborative development mechanism (CDM) to realize the coexistence of the original and new strategies. The process of the collaborative development mechanism is shown in Figure 2. Specifically, the EDA algorithm models the probability distribution using the population generated by the DCS algorithm and generates new individuals accordingly. The DCS utilizes a greedy strategy to select the offspring individuals from the new individuals provided by itself and those generated by the EDA. The individuals used by the DCS may contain individuals generated by the EDA based on the dominant population’s guidance, which allows the DCS approach to utilize the dominant population’s guidance as well. The EDA method utilizes individuals who are not exclusively self-provided, which mitigates the weakening of population diversity.

### 3.4. The Framework of MSDCS

**Figure 3:**
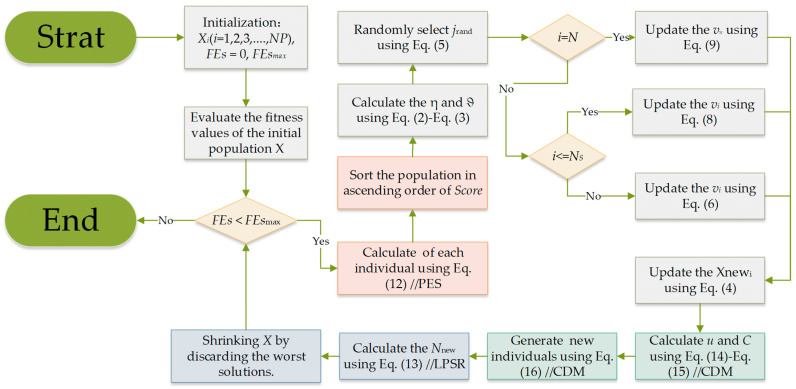
The flowchart of MSDCS.

The MSDCS algorithm is obtained by integrating three improvement strategies into the basic DCS algorithm. PES is designed to sort the populations. LPSR adjusts the number of populations participating in the next iteration at the end of each iteration. CDM replenishes the populations with high-quality individuals. To facilitate the understanding of the execution steps of MSDCS, the corresponding pseudo-code is described in detail in Algorithm 1. In addition, a flowchart depicting the MSDCS algorithm is provided in Figure 3.
**Algorithm 1.** Multi Strategy Differentiated Creative Search (MSDCS)1: Initialization: *X_i_*(*i* = 1, 2, 3, …, *NP*), *FEs* = 0, *FEs_max_*2: Evaluate the fitness values of the initial population *X*3: while (*FEs* < *FEs_max_*) do4: Calculate Score of each individual using Equation (12)//**PES**5: Sort the population in ascending order of Score
6:    for every individual, do7:     Calculate the $\eta_{i}^{t}$ and $\vartheta_{i}^{t}$ using Equations (2) and (3)8:     Randomly select $j_{rand}$ using Equation (5)9:     if *i == N*
10:        Update the $v_{N}$ using Equation (9)11:     else if $i\leq N_{s}$
12:        Update the $v_{i,j}$ using Equation (8)13:     else 14:        Update the $v_{i,j}$ using Equation (6)15:     end if16:     Update the *Xnew*_i_ using Equation (4)17:     *FEs* = *Fes* + 118:    end for19:    Calculate μ and C using Equations (14) and (15)//**CDM**20:    Generate $N_{c}$ new individuals using Equation (16)//**CDM**21:    *FEs* = *Fes* + *N*_c_22:    Calculate the *Nnew* using Equation (13)//**LPSR**23:    Shrinking X by discarding the worst solutions24: end while25: Return best individual

### 3.5. Complexity Analysis of MSDCS

In this section, we analyze the time complexity and space complexity of MSDCS. Time complexity measures the rate at which the algorithm’s execution time increases as the input size grows. It indicates the order of magnitude of the time required as the problem size expands. On the other hand, space complexity measures the rate at which the amount of memory required by the algorithm grows as the input size increases, reflecting the algorithm’s demand for memory resources. Both time complexity and space complexity are critical metrics for evaluating the efficiency of an algorithm.

For the basic DCS algorithm, the time complexity and space complexity of DCS are $O\left(N\times D\times T\right)$ and $O\left(N\times D\right)$, respectively, according to its original literature. In this subsection, T denotes the number of iterations, N represents the population size, and D is the dimensionality of the search space.

For the proposed MSDCS, the three improved strategies do not add an initialization step, so the time complexity of population initialization remains unchanged. In the whole optimization process, the PES strategy does not involve population updating and fitness calculation, which has no effect on the time complexity. The CDM strategy additionally generates $N_{c}$ individuals, and the time complexity of this part is $O\left(N_{c}\times D\times T\right)$. The LPSR strategy ensures that the number of evaluations $N_{L}$ per iteration is less than N, which means that the time complexity is reduced. Therefore, the time complexity of MSDCS is $O\left(N_{c}\times D\times T+N_{L}\times D\times T\right)$. The space complexity of MSDCS can be analyzed based on the main data structures and variables involved in storing the information. The most important variables in the algorithm are the particle position matrix X and the particle fitness matrix. This matrix stores the coordinates of each particle, so its space complexity is $O\left(N\times D\right)$. Other parts of the algorithm, such as the PES and CDM, while updating the individual positions, only require temporary variables to store intermediate results. These operations do not contribute additional space complexity. Therefore, the extra memory required for intermediate calculations does not exceed $O\left(N\times D\right)$. In conclusion, the overall space complexity of MSDCS is $O\left(N\times D\right)$.

## 4. Numerical Experiments Using CEC 2017 Test Suite

In this section, we focus on the performance of the proposed MSDCS algorithm on the CEC2018 test suite with the aim of evaluating its capability in dealing with complex test functions. Section 4.1 describes the CEC2018 benchmark test functions in detail and provides a description of the parameters for the comparison algorithms. In addition, this subsection describes the hardware and software configurations of the experimental platform. Section 4.2 and Section 4.3 show the results of the parameter sensitivity analysis and ablation experiments, respectively, which provide insights into the effects of different parameter settings and strategies on the performance of MSDCS. Finally, in Section 4.4, we provide a comprehensive performance comparison analysis of MSDCS with other comparative algorithms to further validate its effectiveness.

### 4.1. Experiment Setting and Performance Metrics

The hardware configuration used in this experiment includes an AMD R9 7945HX, 2.5 Ghz CPU, 32 GB of memory, and a 4060 GPU. The software configuration consists of the Windows 11 operating system and MATLAB 2023a.

To test the performance of MSDCS, the IEEE CEC 2018 test suite is selected; it has 29 test functions, in which F1 is an unimodal function, F3–F9 are multimodal functions, F10–F19 are hybrid functions, and F20–F29 are composite functions. The unimodal function with a unique global optimal solution is mainly used to evaluate the convergence accuracy and speed of the algorithm in basic optimization scenarios. Multi-peak functions contain multiple local extremes and are used to test the algorithm’s ability to find the global optimal solution as well as its ability to jump out of the local optimum. Hybrid functions, on the other hand, combine multiple local optima and global optima, through which the balance between the algorithm’s ability to explore globally and exploit locally in a complex search space can be tested. Composite functions further increase the complexity and difficulty of the problem and are used to test the algorithm’s ability to solve highly complex and comprehensive problems. In this work, the maximum number of function evaluations is used instead of the maximum number of iterations as a stopping criterion to fairly compare the performance of each algorithm. The dimensions are 10D, 30D, 50D, and 100D, and the search range is [−100, 100]*^D^*. The $FEs_{\max}$ is 1000 times the number of dimensions, which is set to 10,000, 30,000, 90,000, and 100,000, respectively. All experimental results are obtained by running each algorithm independently 30 times. The optimum, mean, and standard deviation of all experiments were recorded, and the data were analyzed using the Friedman test and Wilcoxon rank sum test.

To rigorously assess the performance of the MSDCS algorithm, it was compared against several advanced basic and improved algorithms, including (1) evolution-based algorithms: AE [57], LSHADE [58], and APSM-jSO [59]; (2) physics-based algorithms: RIME [60], RDGMVO [61]; (3) mathematics-based algorithms: QIO [62], MTVSCA [63]; (4) swarm-based algorithms: MRFO [64], EOSMA [65]. Table 1 provides the detailed parameter settings of these comparative algorithms, derived from the respective original literature.

### 4.2. Parameter Sensitivity Analysis

**Figure 4:**
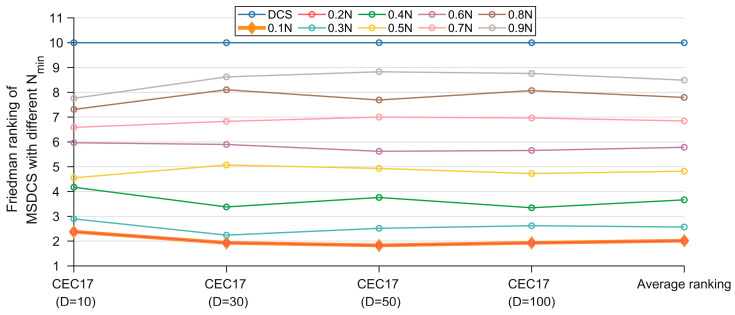
Friedman rankings of MSDCS with different $N_{\min}$ (α = 0.05).

**Figure 5:**
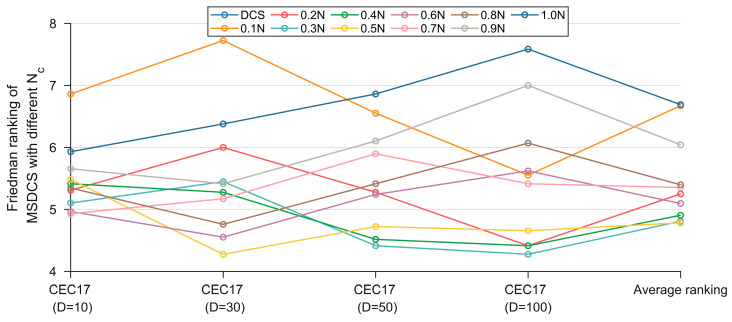
Friedman rankings of MSDCS with different $N_{c}$ (α = 0.05).

The parameter settings have a great impact on the search efficiency of the metaheuristic algorithm. Two important parameters that affect the performance of MSDCS are the minimum population $N_{\min}$ for the LPSR strategy and the population $N_{c}$ for the CDM strategy. Parameter sensitivity is analyzed on the CEC2017 test set with the goal of finding a parameter setting with a relatively high average ranking. Note that the experimental results in this section are based on 30 separate runs. The grid search is conducted. The $N_{\min}$ is searched from 0.1*N* to 0.9*N* with a value interval of 0.1*N*. The $N_{c}$ is searched from 0.1*N* to 1.0*N* with a value interval of 0.1*N*. Table 2 and Table 3 show the statistical results of Friedman’s test using α = 0.05 significance level for $N_{\min}$ and $N_{c}$, respectively, and the best ranking is bold. Figure 4 and Figure 5 show the rankings for different parameter settings.

For $N_{\min}$, we can see that MSDCS achieves the best average ranking when $N_{\min}=0.1N$ or $N_{\min}=0.2N$. Moreover, as $N_{\min}$ decreases, the corresponding rankings increase. This shows that the LPSR strategy can improve the algorithm’s performance by dynamically adjusting the population size. It should be noted that the value of n chosen in this paper is 0.2N, which is because the CDM strategy needs to model the probability distribution through a sufficiently large number of populations, and too small a number cannot represent the population evolutionary trend.

For $N_{c}$, MSDCS with $N_{c}=0.7N$ achieved the best average ranking on 10D. The setting of $N_{c}=0.5N$ allowed MSDCS to reach the best Friedman ranking on 30D. For 50D and 100D, MSDCS performed best overall when $N_{c}=0.3N$. When all four dimensions are considered together, the parameter setting of $N_{c}=0.5N$ ensures that MSDCS has the highest overall average ranking. In conclusion, the parameter settings we chose for MSDCS were $N_{\min}=0.2N$ and $N_{c}=0.5N$.

### 4.3. Ablation Experiment

This subsection discusses the effects of PES, LPSR, and CDM on MSDCM performance. Three MSDCS variants combining a single improvement strategy were developed as shown in Table 4. DCS-PES, DCS-LPSR, and DCS-CDM can be compared with the basic DCS to demonstrate the effectiveness of each strategy. Comparison of DCS-PES, DCS-LPSR, DCS-CDM and MSDCS can be further verified to the promotion between the improvement strategies.

**Figure 6:**
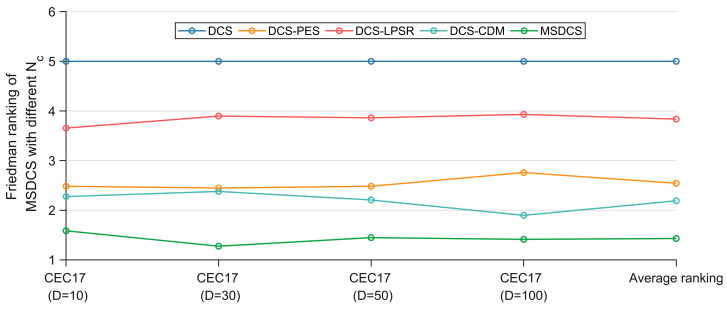
Friedman rankings of MSDCS with different strategy (α = 0.05).

DCS, DCS-PES, DCS-LPSR, DCS-CDM and MSDCS were run independently 30 times in the four-dimensional settings of the CEC2017 test suite and the results were analyzed using Friedman’s test. The original data containing the optimum values, mean values, and standard deviations can be found in Table A1, Table A2, Table A3 and Table A4 in the Appendix A file. Table 5 summarizes the Friedman rankings for DCS, DCS-PES, DCS-LPSR, DCS-CDM, and MSDCS and is visualized in Figure 6. The Friedman test is a nonparametric statistical test used to compare the overall performance of multiple algorithms for significant differences. It determines the performance of an algorithm by ranking its performance on the test set and calculating the average ranking.

According to Table 5, the *p*-value for all four cases is less than 0.05, which indicates that there is a significant difference between DCS, DCS-PES, DCS-LPSR, DCS-CDM, and MSDCS. Based on Table 5 and Figure 6, the discussion is as follows. MSDCS ranks first in all four cases with an overall average ranking of 1.431, followed by DCS-CDM, DCS-PES, and DCS-LPSR, in that order, with the basic DCS coming last. The average ranking of the three DCS variants incorporating a single improvement strategy is better than that of the basic DCS, indicating that the single improvement strategy is effective in enhancing the DCS performance. The enhancement of DCS by PES and CDM is similar at 10D, 30D, and 50D, but CDM enhances the DCS performance more at 100D. The effects of the three strategies on DCS performance are CDM > PES > LPSR in descending order. The MSDCS, incorporating the three improvement strategies, outperforms the DCS variant incorporating a single improvement strategy, which indicates that the three improvement strategies can enhance each other to further enhance the DCS performance.

The excellent performance of the DCS-CDM algorithm is attributed to the guidance of the CDM strategy, which calculates the distribution trend of the dominant population through a Gaussian probability distribution model and thus guides the evolution of the entire population. In addition, the CDM strategy enriches the population diversity by adding individuals to the population that are not new individuals generated based on a single old individual. The PES strategy selects agents by comprehensively evaluating fitness and distance, which allows for a better balance between exploitation and exploration. However, since it does not implement a new search strategy, it does not improve the performance of the DCS as much as the CDM strategy. The LPSR strategy ensures that convergence can be accelerated at a later stage by shrinking the population, but, at the same time, this measure somewhat weakens the population diversity. Each of the three strategies has its own focus and at the same time complements the other to realize the enhancement of MSDCS.

### 4.4. Comparison with Other Advanced Algorithms

After the parameter sensitivity analysis and ablation experiments, we further perform a full comparison using the advanced improved and basic algorithms and the proposed MSDCS in the CEC2017 test set. In this subsection, this paper mainly analyzes the experimental results using the Friedman test and Wilcoxon rank sum test. The complete experimental results obtained by MSDCS and the comparison algorithms are summarized in Table A5, Table A6, Table A7 and Table A8 in Appendix A, including the best value (Best), the average value (Ave), the standard deviation (Std), and the rank. As the Friedman test and Wilcoxon test analyze the performance of MSDCS and the comparison algorithms as a whole, this paper uses heat maps to initially show the mean-based rankings of MSDCS and the competition algorithms on each function.

**Figure 7:**
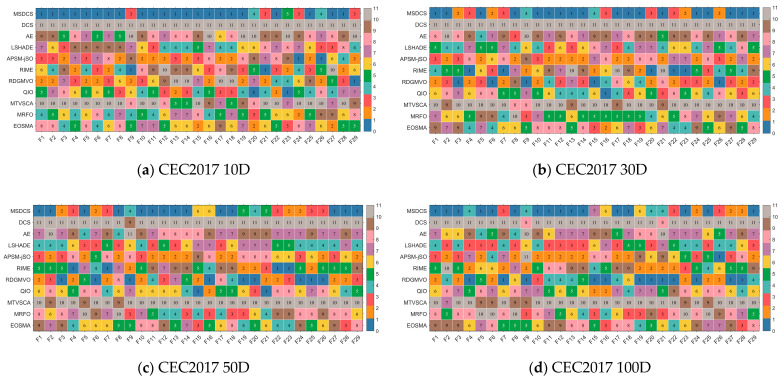
The ranking heatmaps of MSDCS and comparison algorithms solving CEC2017 test suite.

The ranking of MSDCS and the comparison algorithms on each function in different dimensions is visualized in Figure 7. In Figure 7, MSDCS ranks first with 22 functions on 10D, performs best with 18 functions on 30D, provides the best solution with 15 functions on 50D, and achieves the top ranking with 16 functions on 100D. That is, MSDCS achieved the best rankings in more than half of the 29 CEC2017 test functions under different dimensions, which tentatively shows the superiority of MSDCS.

**Figure 8:**
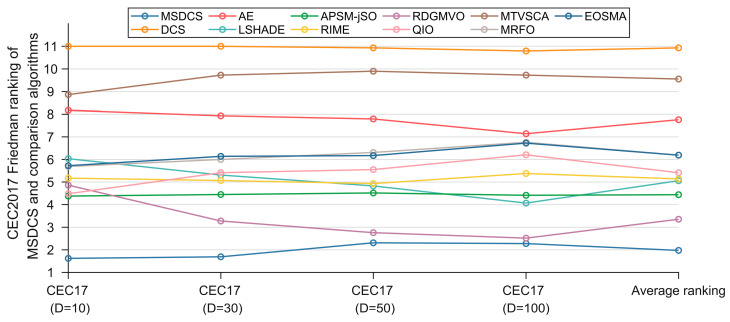
Friedman rankings of MSDCS and comparison algorithms (α = 0.05).

Table 6 summarizes the Friedman test results for MSDCS, DCS, AE, LSHADE, APSM-jSO, RIME, RDGMVO, QIO, MTVSCA, MRFO, and EOSMA, and the corresponding rankings are displayed in Figure 8. In Figure 8, the rankings of all the algorithms are kept stable across dimensions, which suggests that the algorithms that participate in the experiments have good robustness. The proposed MSDCS is ranked first in all four cases, and DCS is ranked last. This fully reflects the advantage of MSDCS over DCS. MSDCS and RDGMVO are ranked similarly on 50D and 100D, which is consistent with the analysis results in Figure 7. According to Table 6, the Friedman *p*-value for all four dimensions is less than 0.05, which means that there is a significant difference between MSDCS and DCS, AE, LSHADE, APSM-jSO, RIME, RDGMVO, QIO, MTVSCA, MRFO, and EOSMA. The following provides specific Friedman test results from Table 6.

For CEC2017 10D, MSDCS ranks in the first place, followed by APSM-jSO, QIO, RDGMVO, RIME, MRFO, EOSMA, LSHADE, AE, MTVSCA, and DCS. This shows that MSDCS is outpacing the competition algorithm when solving CEC2017 10D functions.

For CEC2017 30D, MSDCS ranks in the first place, followed by RDGMVO, APSM-jSO, RIME, LSHADE, QIO, MRFO, EOSMA, AE, MTVSCA, and DCS. This shows that MSDCS is outpacing the competition algorithm when solving CEC2017 50D functions.

For CEC2017 50D, MSDCS ranks in the first place, followed by RDGMVO, APSM-jSO, LSHADE, RIME, QIO, EOSMA, MRFO, AE, MTVSCA, and DCS. This shows that MSDCS is outpacing the competition algorithm when solving CEC2017 50D functions.

For CEC2017 100D, MSDCS ranks in the first place, followed by RDGMVO, LSHADE, APSM-jSO, RIME, QIO, EOSMA, MRFO, AE, MTVSCA, and DCS. This shows that MSDCS is outpacing the competition algorithm when solving CEC2017 100D functions.

**Figure 9:**
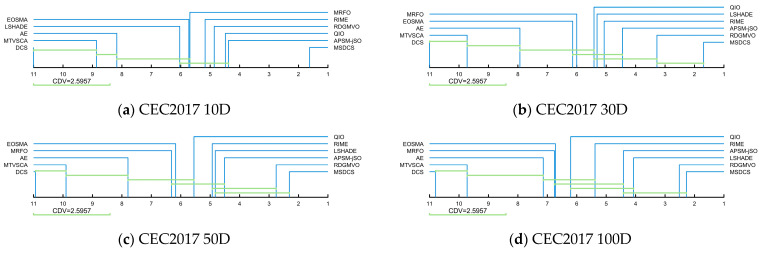
The Nemenyi test results of MSEDO and comparison algorithms.

The Friedman test only indicates whether there is a difference between MSDCS and the comparison algorithms, but it does not formalize the difference between MSDCS and each algorithm. Based on the results of the Friedman test, the Nemenyi test was used as a post hoc test. Figure 1 illustrates the magnitude of the differences between MSDCS and the comparison algorithms, where the algorithms that are connected by lines indicate that there are no significant differences between them. From Figure 9, MSDCS differs from all comparison algorithms on 10D. As for RDGMVO, MSDCS has no significant difference in the remaining three dimensions. For APSM-jSO and LSHADE, MSDCS differs on 30D and has no difference on 50D and 100D. For all other algorithms, MSDCS shows significant superiority. In conclusion, MSDCS has significant advantages and is a promising DCS variant.

**Figure 10:**
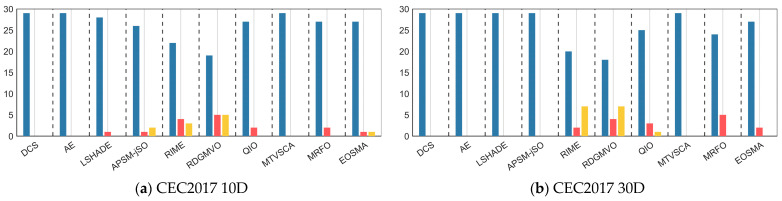

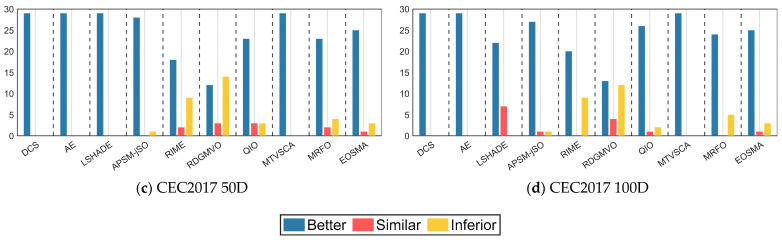
Visualization of Wilcoxon rank sum test results of MSDCS and comparison algorithms (α = 0.05).

This was followed by a Wilcoxon rank sum test to examine the differences between MSDCS and all comparison algorithms for each function. Table 7 summarizes the statistics for MSDCS, DCS, AE, LSHADE, APSM-jSO, RIME, RDGMVO, QIO, MTVSCA, MRFO, and EOSMA, where the number of symbols “+” denotes the number of functions for which MSDCS outperforms the comparative algorithms. The number of “−” symbols indicates the number of functions for which MSDCS is inferior to the comparison algorithm. The number of “=” indicates the number of functions for which MSDCS and the comparison algorithm have similar performance. Figure 10 shows the number of functions for which MSDCS is better/similar/inferior to the comparison algorithm. From the “Total” in the last column of Table 7, it can be seen that MSDCS significantly outperforms at least half of the functions when compared with different algorithms, which indicates that the overall performance of MSDCS is better than that of other comparative algorithms. In 10D, MSDCS only underperforms the other algorithms when compared with APSM-jSO, RIME, RDGMVO, and EOSMA, but at least 19 functions are better than these algorithms. In 30D, MSDCS only loses to RIME, RDGMVO, and QIO in some of the functions, but outperforms each of them in more functions (at least 18). In higher-dimensional functions (50D/100D), MSDCS performance drops slightly. MSDCS scores against each other in comparisons with APSM-jSO, RIME, RDGMVO, and QIO. For 50D, MSDCS achieves fewer “+” than “−” in the comparison with RDGMVO. However, combining the Friedman test and heat map analysis, MSDCS is the better performer in all three functions, in which MSDCS and RDGMVO perform similarly. Therefore, we can still conclude that the overall performance of MSDCS is superior to that of RDGMVO for the 50D functions. Notably, MSDCS achieves the success of all the functions under all four dimensions in comparison with DCS. In conclusion, the Wilcoxon rank sum test reconfirms the great performance of MSDCS.

## 5. Numerical Experiments Using Engineering Optimization Problem

In this section, we apply MSDCS to solve engineering optimization problems with the aim of evaluating its potential for solving real-world optimization problems. The engineering optimization problems contain the wireless sensor network coverage problem and the pressure vessel design problem. The parameter settings for all algorithms involved in the following experiments of this section are the same as in Table 1.

### 5.1. Wireless Sensor Network Coverage Problem

Wireless sensor networks (WSNs) have become more popular than ever. WSNs represent an emerging computing and networking paradigm that can be defined as networks composed of miniature, small, inexpensive, and highly intelligent devices known as sensor nodes. In wireless sensor network node deployment, we deploy N wireless sensor nodes in a two-dimensional planar region. The set of homogeneous wireless sensor nodes is $S=\{s_{1},s_{2},s_{3},\ldots ,s_{i},\ldots ,s_{N}\}$. Each node can obtain its position and has an identical sensing radius $R_{s}$. We assume the monitoring area is a 2D rectangular area of L×W. The set of monitoring nodes is $M=\{m_{1},m_{2},m_{3},\ldots ,m_{j},\ldots ,M_{L\times W}\}$. $\left(x_{i},y_{i}\right)$ denotes the coordinates of each sensor node $s_{i}$. $\left(x_{j},y_{j}\right)$ denotes the coordinates of each monitoring node $m_{j}$. The Euclidean distance between the two nodes is as below:

(18) $$d(s_{i},m_{j})=\sqrt{{\left(x_{i}-x_{j}\right)}^{2}+{\left(y_{i}-y_{j}\right)}^{2}}$$

In this paper, we use a Boolean 0–1 model to determine whether a sensor node can detect a monitoring node or not. The probability of monitoring point $m_{j}$ being sensed by node $s_{i}$ is defined as follows:

(19) $$p_{c}(s_{i},m_{j})=\left\{\begin{array}{l} 1,if\;\;d(s_{i},m_{j})\leq R_{s}\\ 0,otherwise \end{array}\right.$$

The all-sensor coverage rate $C_{r}$ can be obtained from Equation (20).

(20) $$C_{r}=\frac{\sum_{j=1}^{L\times W}p_{c}(s_{i},m_{j})}{L\times W}$$

**Figure 11:**
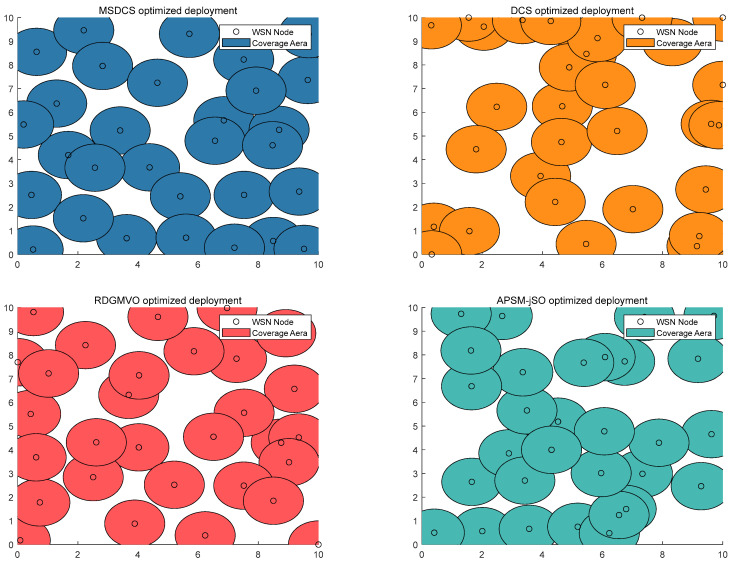
Node deployment of MSDCS and its competitors.

**Figure 12:**
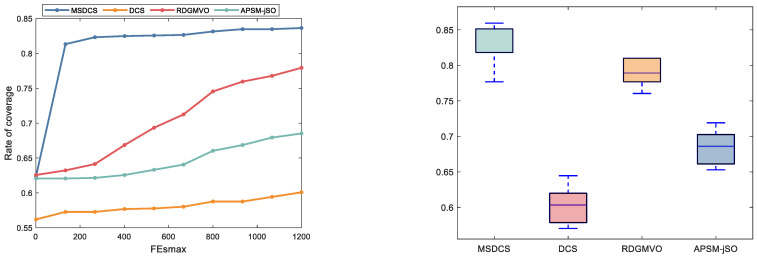
Convergence graph and boxplots of MSDCS and its competitors.

Figure 11 presents the visual representation of the coverage area for different algorithms in solving the wireless network coverage problem. Figure 12 displays the convergence curves and boxplots for the same problem, showing the coverage area for different algorithms. It is evident that MSDCS achieves the largest network coverage area, outperforming the DCS, RDGMVO, and APSM-jSO. The experimental results presented in Table 8 compare the performance of various algorithms in optimizing sensor wireless network coverage. The MSDCS algorithm achieves the highest coverage value of 85.95%, demonstrating its superior ability to maximize sensor coverage. This result highlights MSDCS’s exceptional efficiency in optimizing network coverage compared to other algorithms. The RDGMVO algorithm follows closely behind with a value of 80.99%, showing competitive performance in terms of coverage efficiency. The APSM-jSO algorithm achieves a coverage value of 71.90%, performing slightly better than the DCS algorithm, which achieved a value of 64.46%. The convergence curves show that the MSDCS algorithm is able to find the best coverage scheme quickly. The boxplot shows that MSDCS has the best robustness and provides a more centralized distribution of solutions. These results confirm that the proposed MSDCS is the most efficient approach to solving the sensor network coverage problem, outperforming other compared algorithms.

### 5.2. Constrained Engineering Optimization

**Figure 13:**
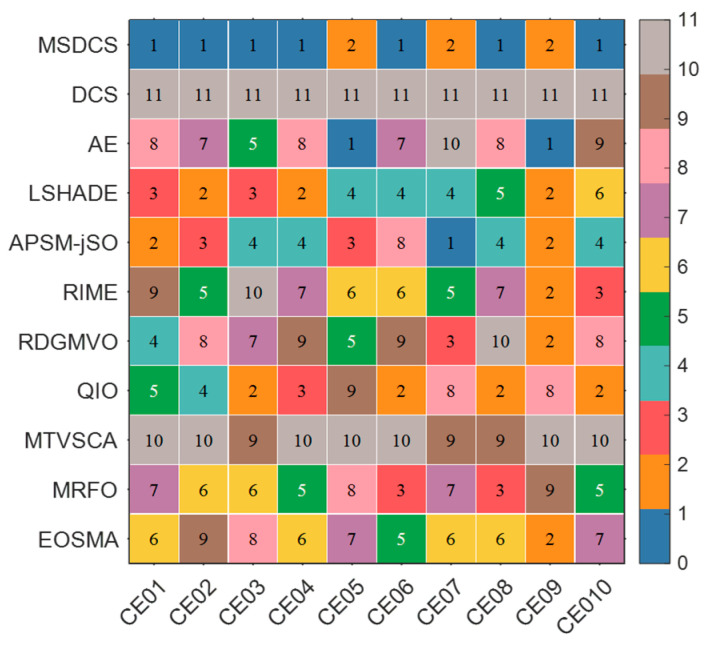
The ranking heatmaps of MSDCS and comparison algorithms solving constrained engineering optimization problems.

In this subsection, we verify the performance of MSDCS through 10 constrained optimization problems. The details of these constrained problems are displayed in Table 9. When applying meta-heuristic algorithms to solve constrained optimization problems, the handling of constraints is crucial. Commonly used methods include the penalty function method, repair strategy, and rule-based constraint processing. The penalty function method converts the degree of constraint violation into a penalty term to be added to the objective function; the repair strategy repairs the infeasible solution into a feasible solution; and the rule-based constraint processing utilizes the problem rules to filter or adjust the solution. This paper adopts the penalty function method to convert a constrained optimization problem into an unconstrained optimization problem. That is, a large penalty value is imposed on the solution when it violates the constraints. Table 10 summarizes the results obtained by MSDCS, DCS, AE, LSHADE, APSM-jSO, RIME, RDGMVO, QIO, MTVSCA, MRFO, and EOSMA for solving the constrained optimization problems and visualizes the ranking based on the average values as shown in Figure 13. According to Figure 13, MSEDO provided the best solution on seven constraint problems and the second-best solution on three constraint problems. In sharp contrast, DCS ranks last on all problems. This indicates that the proposed improvement strategies are effective in enhancing the performance of DCS and providing MSEDO with the ability to solve real-world problems.

## 6. Conclusions

This paper presents a DCS algorithm variant, called MSDCS, which achieves DCS performance improvement by integrating three improvement strategies. Firstly, a collaborative development mechanism is proposed to organically integrate the estimation allocation algorithm and DCS. Through the guiding effect of the dominant population, it makes up for the defects that the DCS algorithm has, such as an insufficient exploratory ability and the fact that it can easily fall into local optimization. Secondly, a new population evaluation strategy is proposed to realize the coordinated transition of development and exploration through the comprehensive evaluation of adaptation and distance. Finally, a linear population size reduction strategy is integrated into the distributed control system to enhance the exploration ability and extensively search the solution space by maintaining a large population size at the initial stage. The proposed MSDCS algorithm is validated on the CEC2017 test set, a wireless sensor network coverage problem, and an engineering-constrained optimization problem, and the following conclusions are presented.

On the CEC2017 test suite, we obtained the optimal parameter settings through parameter sensitivity analysis and verified the effectiveness of the improved strategies through ablation experiments. The excellent performance of MSDCS is validated by comparing it with other advanced basic and improved algorithms and applying Friedman’s test and Wilcoxon rank sum test. On the wireless sensor network coverage problem, MSDCS provides the best coverage solution with an 85.95% coverage rate. In addition, MSDCS performs well on 10 engineering-constrained optimization problems, ranking first in 7 of them. In summary, the MSDCS proposed in this paper is a promising metaheuristic algorithm variant. Certainly, MSDCS has some limitations. Firstly, the parameters of MSDCS need to be determined manually, and its generalized optimal parameter settings on a wider range of problems need further research. Secondly, MSDCS does not perform as well on high-dimensional problems as it does on low-dimensional problems, which may be caused by the mutual constraints between the too small number of populations at the later stage and the sufficient number of populations required by the synergistic development mechanism. This suggests that MSDCS needs to further improve its ability to solve high-dimensional problems. In addition, the optimal parameters need to be determined experimentally, and such fixed parameters may weaken the algorithm’s search capability, leading to premature convergence and, in some cases, to local optima. Finally, it is necessary to develop a multi-objective version of MSDCS, which is due to the fact that real-world optimization problems often have multiple objectives to satisfy and are difficult to achieve at the same time.

## Figures and Tables

**Table 1:** Parameters setting of MSDCS and other selected algorithms.

Algorithm	Parameters Setting
MSDCS	$\begin{array}{l} p_{c}=0.5,\gamma =0.6,N_{s}=\max\left\{6,round\left(\frac{2N}{3+3\sqrt{5}}\right)\right\}\\ N_{\min}=\max\left\{N_{s}+4,round\left(0.2N\right)\right\},N_{c}=0.5N \end{array}$
DCS	m=3
AE [57]	w=4
LSHADE [58]	$F=0.5,Cr=0.5,p=0{.11,N}_{\min}=4$
APSM-jSO [59]	k=3,F=0.3,CR=0.8,H=6
RIME [60]	W=5
RDGMVO [61]	$w_{\max}=1,w_{\min}=0.2,p=0.6,\alpha =0.1$
QIO [62]	$\alpha_{1}=0.7,\alpha_{2}=0.15,w=3$
MTVSCA [63]	λ=0.25,n=20,a=2
MRFO [64]	S=2
EOSMA [65]	$V=1,a_{1}=2,a_{2}=1,GP=0.5,z=0.6,q=0.2$

**Table 2:** Rankings of MSDCS with different $N_{\min}$ (α = 0.05).

Dimension	DCS	*0.1N*	*0.2N*	*0.3N*	*0.4N*	*0.5N*	*0.6N*	*0.7N*	*0.8N*	*0.9N*
D = 10	10.000	2.379	2.379	2.897	4.172	4.552	5.966	6.586	7.310	7.759
D = 30	10.000	1.931	1.931	2.241	3.379	5.069	5.897	6.828	8.103	8.621
D = 50	10.000	1.828	1.828	2.517	3.759	4.931	5.621	7.000	7.690	8.828
D = 100	10.000	1.931	1.931	2.621	3.345	4.724	5.655	6.966	8.069	8.759
Average ranking	10.000	2.017	2.017	2.569	3.664	4.819	5.784	6.845	7.793	8.491

**Table 3:** Rankings of MSDCS with different $N_{c}$ (α = 0.05).

Dimension	DCS	*0.1N*	*0.2N*	*0.3N*	*0.4N*	*0.5N*	*0.6N*	*0.7N*	*0.8N*	*0.9N*	*0.10N*
D = 10	11.000	6.862	5.310	5.103	5.414	5.483	4.966	4.931	5.345	5.655	5.931
D = 30	11.000	7.724	6.000	5.448	5.276	4.276	4.552	5.172	4.759	5.414	6.379
D = 50	11.000	6.552	5.276	4.414	4.517	4.724	5.241	5.897	5.414	6.103	6.862
D = 100	11.000	5.552	4.414	4.276	4.414	4.655	5.621	5.414	6.069	7.000	7.586
Average ranking	11.000	6.672	5.250	4.810	4.905	4.784	5.095	5.353	5.397	6.043	6.690

**Table 4:** Descriptions of MSDCS with different strategies.

Strategy	DCS	DCS-PES	DCS-LPSR	DCS-CDM	MSDCS
PES	No	Yes	No	No	Yes
LPSR	No	No	Yes	No	Yes
CDM	No	No	No	Yes	Yes

**Table 5:** Rankings of MSDCS with different strategy (α = 0.05).

Test suite	Dimension	DCS	DCS-PES	DCS-LPSR	DCS-CDM	MSDCS	*p*-Value
CEC 2017	10	5.000	2.483	3.655	2.276	1.586	2.79E-17
	30	5.000	2.448	3.897	2.379	1.276	2.37E-20
	50	5.000	2.483	3.862	2.207	1.448	2.55E-19
	100	5.000	2.759	3.931	1.897	1.414	7.92E-21
Average ranking		5.000	2.543	3.836	2.190	1.431	N/A
Ranking		5	3	4	2	1	N/A

**Table 6:** Rankings of MSDCS and comparison algorithms (α = 0.05).

Algorithm	CEC-2017 Test Suite					
	10D	30D	50D	100D	Average Ranking	Rank
MSDCS	1.621	1.690	2.310	2.276	1.974	1
DCS	11.000	11.000	10.931	10.793	10.931	11
AE	8.172	7.931	7.793	7.138	7.759	9
LSHADE	6.034	5.310	4.828	4.069	5.060	4
APSM-jSO	4.379	4.448	4.517	4.414	4.440	3
RIME	5.172	5.069	4.931	5.379	5.138	5
RDGMVO	4.862	3.276	2.759	2.517	3.353	2
QIO	4.483	5.414	5.552	6.207	5.414	6
MTVSCA	8.862	9.724	9.897	9.724	9.552	10
MRFO	5.690	6.000	6.310	6.759	6.190	7
EOSMA	5.724	6.138	6.172	6.724	6.190	7
Friedman P-value	*4.10E-31*	8.69E-36	2.49E-35	2.39E-35	N/A	N/A

**Table 7:** The Wilcoxon rank sum test results of MSDCS and comparison algorithms (α = 0.05).

MSDCS vs.	10D (+/=/−)	30D (+/=/−)	50D (+/=/−)	100D (+/=/−)	Total (+/=/−)
DCS	29/0/0	29/0/0	29/0/0	29/0/0	116/0/0
AE	29/0/0	29/0/0	29/0/0	29/0/0	116/0/0
LSHADE	28/1/0	29/0/0	29/0/0	22/7/0	108/8/0
APSM-jSO	26/1/2	29/0/0	28/0/1	27/1/1	108/2/4
RIME	22/4/3	20/2/7	18/2/9	20/0/9	80/8/28
RDGMVO	19/5/5	18/4/7	12/3/14	13/4/12	62/16/38
QIO	27/2/0	25/3/1	23/3/3	26/1/2	101/9/6
MTVSCA	29/0/0	29/0/0	29/0/0	29/0/0	116/0/0
MRFO	27/2/0	24/5/0	23/2/4	24/0/5	95/9/9
EOSMA	27/1/1	27/2/0	25/1/3	25/1/3	104/5/7

**Table 8:** The statistical results of MSDCS and competition algorithms for solving WSN.

**Algorithm**	**Best**	**Mean**	**Std**
MSDCS	85.95%	83.64%	0.026
DCS	64.46%	60.08%	0.025
RDGMVO	80.99%	78.93%	0.018
APSM-jSO	71.90%	68.51%	0.022

**Table 9:** Ten constrained engineering optimization problems.

Problem	Name	D
CE01	Tension/compression spring design problem	3
CE02	Pressure vessel design problem	4
CE03	Three-bar truss design problem	2
CE04	Welded beam design problem	4
CE05	Speed reducer design problem	7
CE06	Gear train design problem	4
CE07	Rolling element bearing design	10
CE08	Cantilever beam design problem	5
CE09	Multiple disk clutch brake design problem	5
CE10	Step-cone pulley problem	5

**Table 10:** The statistics results of MSDCS and competition algorithms solving constrained engineering optimization problems.

Function	Index	MSDCS	DCS	AE	LSHADE	APSM-jSO	RIME	RDGMVO	QIO	MTVSCA	MRFO	EOSMA
CE1	Best	1.2671E-02	2.4083E-02	1.3191E-02	1.2907E-02	1.2871E-02	1.3529E-02	1.3044E-02	1.2921E-02	1.3906E-02	1.3057E-02	1.2846E-02
	Mean	1.2860E-02	7.3531E+03	1.4128E-02	1.3296E-02	1.3054E-02	1.6235E-02	1.3356E-02	1.3383E-02	1.6551E-02	1.3777E-02	1.3695E-02
	Std	2.8359E-04	1.6442E+04	1.0954E-03	3.2821E-04	1.4931E-04	2.9673E-03	4.1936E-04	4.1607E-04	2.4984E-03	7.4353E-04	9.4204E-04
	Rank	1	11	8	3	2	9	4	5	10	7	6
CE2	Best	5.8701E+03	1.7715E+05	6.6293E+03	6.3630E+03	6.0647E+03	6.0556E+03	7.6793E+03	6.3236E+03	1.2028E+04	6.5909E+03	7.1900E+03
	Mean	5.9511E+03	3.2762E+05	8.0280E+03	6.5037E+03	6.5380E+03	6.9088E+03	8.3425E+03	6.8454E+03	1.3060E+04	7.0169E+03	8.3431E+03
	Std	1.6259E+02	1.9473E+05	1.1292E+03	2.4059E+02	4.8136E+02	7.6999E+02	1.1312E+03	3.2487E+02	1.1049E+03	5.8863E+02	1.0649E+03
	Rank	1	11	7	2	3	5	8	4	10	6	9
CE3	Best	2.6389E+02	2.6395E+02	2.6391E+02	2.6390E+02	2.6390E+02	2.6394E+02	2.6389E+02	2.6389E+02	2.6405E+02	2.6389E+02	2.6390E+02
	Mean	2.6389E+02	2.6468E+02	2.6393E+02	2.6390E+02	2.6392E+02	2.6442E+02	2.6394E+02	2.6390E+02	2.6433E+02	2.6394E+02	2.6402E+02
	Std	2.8422E-14	1.0184E+00	1.4357E-02	5.1566E-03	2.7481E-02	7.0931E-01	5.5071E-02	2.6617E-03	2.9947E-01	4.1346E-02	1.6598E-01
	Rank	1	11	5	3	4	10	7	2	9	6	8
CE4	Best	1.6928E+00	2.2522E+00	1.8755E+00	1.7093E+00	1.7255E+00	1.7528E+00	1.8060E+00	1.7362E+00	1.8941E+00	1.7390E+00	1.7520E+00
	Mean	1.6928E+00	3.1607E+00	1.9597E+00	1.7362E+00	1.7744E+00	1.9554E+00	1.9708E+00	1.7493E+00	2.0044E+00	1.7759E+00	1.8056E+00
	Std	6.0366E-07	7.2634E-01	5.6745E-02	2.1821E-02	7.5693E-02	2.3237E-01	2.3252E-01	1.4943E-02	8.2997E-02	4.0763E-02	4.0271E-02
	Rank	1	11	8	2	4	7	9	3	10	5	6
CE5	Best	2.9936E+03	3.1674E+03	2.8842E+03	2.9955E+03	2.9937E+03	3.0006E+03	3.0000E+03	3.0179E+03	3.0581E+03	3.0126E+03	3.0136E+03
	Mean	2.9936E+03	3.6127E+03	2.9817E+03	2.9964E+03	2.9937E+03	3.0081E+03	3.0054E+03	3.0395E+03	3.0836E+03	3.0205E+03	3.0196E+03
	Std	1.2705E-08	5.4941E+02	8.1403E+01	5.7719E-01	4.6451E-02	1.4537E+01	3.5675E+00	1.7883E+01	2.8303E+01	6.8185E+00	4.7228E+00
	Rank	2	11	1	4	3	6	5	9	10	8	7
CE6	Best	2.7009E-12	6.5257E-08	2.7009E-12	2.7009E-12	2.7009E-12	2.3078E-11	3.0676E-10	2.7009E-12	9.7457E-10	2.3078E-11	2.3078E-11
	Mean	2.5107E-10	2.9280E-04	1.6136E-09	1.3787E-09	1.8025E-09	1.6091E-09	1.8218E-09	6.9316E-10	1.5824E-08	1.0321E-09	1.4391E-09
	Std	5.2127E-10	2.6088E-04	1.0773E-09	9.9970E-10	2.8168E-09	1.6964E-09	1.6550E-09	6.3136E-10	3.1042E-08	9.6274E-10	1.2581E-09
	Rank	1	11	7	4	8	6	9	2	10	3	5
CE7	Best	-2.4358E+05	-1.2042E+05	-1.1897E+03	-2.4358E+05	-2.4358E+05	-2.4358E+05	-2.4358E+05	-2.3030E+05	-2.2659E+05	-2.3612E+05	-2.4144E+05
	Mean	-2.4358E+05	1.5159E+06	-1.0860E+03	-2.4356E+05	-2.4358E+05	-2.4314E+05	-2.4358E+05	-2.2349E+05	-2.2243E+05	-2.3002E+05	-2.4025E+05
	Std	5.8147E-07	1.8087E+06	5.8056E+01	2.7780E+01	1.2992E-09	4.4409E+02	5.3962E+00	8.2081E+03	4.6563E+03	7.9789E+03	8.0731E+02
	Rank	2	11	10	4	1	5	3	8	9	7	6
CE8	Best	1.3400E+00	2.0342E+00	1.4263E+00	1.3467E+00	1.3511E+00	1.3525E+00	1.4144E+00	1.3409E+00	1.4359E+00	1.3426E+00	1.3554E+00
	Mean	1.3400E+00	3.1656E+00	1.4467E+00	1.3601E+00	1.3577E+00	1.4106E+00	1.6551E+00	1.3430E+00	1.5526E+00	1.3496E+00	1.3609E+00
	Std	1.2511E-07	1.1175E+00	1.9849E-02	1.4979E-02	7.8764E-03	4.2675E-02	2.0798E-01	2.9635E-03	9.4868E-02	9.2797E-03	4.3679E-03
	Rank	1	11	8	5	4	7	10	2.0000E+00	9.0000E+00	3.0000E+00	6.0000E+00
CE9	Best	3.9247E+08	3.9247E+08	2.5199E+08	3.9247E+08	3.9247E+08	3.9247E+08	3.9247E+08	3.9247E+08	3.9247E+08	3.9247E+08	3.9247E+08
	Mean	3.9247E+08	3.9474E+08	2.5245E+08	3.9247E+08	3.9247E+08	3.9247E+08	3.9247E+08	3.9247E+08	3.9247E+08	3.9247E+08	3.9247E+08
	Std	0.0000E+00	4.5209E+06	8.8091E+05	0.0000E+00	0.0000E+00	0.0000E+00	0.0000E+00	9.2258E-02	1.8781E+03	3.4870E-01	0.0000E+00
	Rank	2	11	1	2	2	2	2	8	10	9	2
CE10	Best	1.6086E+01	1.7337E+04	2.2769E+01	1.7146E+01	1.6594E+01	1.6282E+01	1.6886E+01	1.6576E+01	7.8319E+01	1.6735E+01	1.7144E+01
	Mean	1.6086E+01	7.0920E+08	8.0915E+01	1.7397E+01	1.7053E+01	1.6967E+01	1.9526E+01	1.6696E+01	1.4682E+02	1.7109E+01	1.7557E+01
	Std	4.8761E-07	1.3571E+09	1.0977E+02	3.2555E-01	4.5030E-01	5.6418E-01	3.9405E+00	1.1886E-01	7.2506E+01	3.1909E-01	4.4901E-01
	Rank	1	11	9	6	4	3	8	2	10	5	7

## Data Availability

Data will be made available on request.

## Appendix A

**Table A1:** The statistical results of MSDCS with different strategies for solving CEC2017 (D = 10).

Function	Index	DCS	DCS-PES	DCS-LPSR	DCS-CDM	MSDCS	Function	Index	DCS	DCS-PES	DCS-LPSR	DCS-CDM	MSDCS
F1	Best	5.749E+09	1.002E+02	1.730E+06	1.589E+02	1.000E+02	F16	Best	1.812E+03	1.731E+03	1.739E+03	1.706E+03	1.708E+03
	Mean	1.416E+10	1.006E+02	6.761E+06	1.959E+03	1.000E+02		Mean	2.139E+03	1.741E+03	1.750E+03	1.722E+03	1.727E+03
	Std	4.455E+09	3.216E-01	3.158E+06	2.124E+03	1.786E-04		Std	1.598E+02	6.389E+00	7.504E+00	9.146E+00	8.155E+00
F2	Best	2.182E+04	3.000E+02	1.777E+03	3.000E+02	3.000E+02	F17	Best	2.449E+06	1.802E+03	6.754E+03	1.803E+03	1.800E+03
	Mean	4.706E+04	3.000E+02	8.772E+03	3.003E+02	3.000E+02		Mean	2.549E+08	1.810E+03	1.515E+04	1.824E+03	1.805E+03
	Std	1.740E+04	9.349E-06	3.893E+03	3.293E-01	2.735E-09		Std	2.261E+08	6.262E+00	6.760E+03	9.643E+00	7.111E+00
F3	Best	7.774E+02	4.002E+02	4.054E+02	4.005E+02	4.001E+02	F18	Best	2.783E+03	1.901E+03	1.942E+03	1.901E+03	1.900E+03
	Mean	1.774E+03	4.011E+02	4.120E+02	4.024E+02	4.007E+02		Mean	8.309E+06	1.903E+03	2.355E+03	1.903E+03	1.901E+03
	Std	6.895E+02	5.480E-01	5.797E+00	7.140E-01	4.802E-01		Std	8.178E+06	6.232E-01	5.995E+02	1.133E+00	5.817E-01
F4	Best	5.899E+02	5.098E+02	5.109E+02	5.115E+02	5.103E+02	F19	Best	2.263E+03	2.015E+03	2.021E+03	2.005E+03	2.008E+03
	Mean	6.366E+02	5.207E+02	5.234E+02	5.170E+02	5.162E+02		Mean	2.375E+03	2.034E+03	2.038E+03	2.014E+03	2.020E+03
	Std	2.293E+01	5.161E+00	4.691E+00	3.418E+00	3.861E+00		Std	5.961E+01	8.083E+00	6.878E+00	6.658E+00	6.720E+00
F5	Best	6.465E+02	6.000E+02	6.004E+02	6.000E+02	6.000E+02	F20	Best	2.278E+03	2.200E+03	2.204E+03	2.200E+03	2.200E+03
	Mean	6.786E+02	6.000E+02	6.009E+02	6.000E+02	6.000E+02		Mean	2.406E+03	2.271E+03	2.230E+03	2.256E+03	2.264E+03
	Std	1.370E+01	1.867E-03	2.781E-01	2.636E-03	3.299E-05		Std	4.711E+01	6.302E+01	4.716E+01	6.132E+01	6.064E+01
F6	Best	8.880E+02	7.262E+02	7.335E+02	7.228E+02	7.203E+02	F21	Best	2.557E+03	2.200E+03	2.267E+03	2.200E+03	2.300E+03
	Mean	9.093E+02	7.329E+02	7.499E+02	7.326E+02	7.281E+02		Mean	3.554E+03	2.297E+03	2.304E+03	2.296E+03	2.300E+03
	Std	1.523E+01	4.019E+00	6.364E+00	5.188E+00	4.288E+00		Std	3.922E+02	1.843E+01	1.082E+01	2.376E+01	5.324E-01
F7	Best	8.570E+02	8.075E+02	8.144E+02	8.093E+02	8.079E+02	F22	Best	2.736E+03	2.610E+03	2.617E+03	2.602E+03	2.604E+03
	Mean	9.033E+02	8.210E+02	8.264E+02	8.169E+02	8.165E+02		Mean	2.796E+03	2.619E+03	2.625E+03	2.614E+03	2.613E+03
	Std	1.358E+01	4.894E+00	5.167E+00	3.665E+00	4.026E+00		Std	2.490E+01	4.661E+00	3.787E+00	4.373E+00	4.436E+00
F8	Best	2.591E+03	9.000E+02	9.036E+02	9.000E+02	9.000E+02	F23	Best	2.846E+03	2.500E+03	2.515E+03	2.500E+03	2.500E+03
	Mean	3.425E+03	9.000E+02	9.109E+02	9.000E+02	9.000E+02		Mean	2.932E+03	2.739E+03	2.571E+03	2.702E+03	2.722E+03
	Std	4.504E+02	6.786E-07	4.790E+00	8.294E-02	2.978E-12		Std	4.990E+01	4.545E+01	6.942E+01	9.186E+01	6.047E+01
F9	Best	2.458E+03	1.499E+03	1.709E+03	1.230E+03	1.437E+03	F24	Best	2.986E+03	2.898E+03	2.906E+03	2.898E+03	2.898E+03
	Mean	3.058E+03	2.068E+03	2.049E+03	1.742E+03	1.882E+03		Mean	3.810E+03	2.924E+03	2.923E+03	2.926E+03	2.927E+03
	Std	3.212E+02	1.870E+02	1.626E+02	2.008E+02	1.643E+02		Std	4.016E+02	2.295E+01	1.407E+01	2.328E+01	2.232E+01
F10	Best	1.465E+03	1.104E+03	1.137E+03	1.102E+03	1.103E+03	F25	Best	3.845E+03	2.900E+03	2.783E+03	2.900E+03	2.900E+03
	Mean	7.862E+03	1.106E+03	1.164E+03	1.105E+03	1.104E+03		Mean	4.601E+03	2.900E+03	2.931E+03	2.902E+03	2.900E+03
	Std	5.270E+03	1.243E+00	1.740E+01	1.684E+00	8.064E-01		Std	3.360E+02	4.062E-04	3.732E+01	8.608E+00	2.980E-07
F11	Best	1.464E+08	1.230E+03	3.190E+05	2.978E+03	1.200E+03	F26	Best	3.168E+03	3.090E+03	3.093E+03	3.089E+03	3.090E+03
	Mean	1.071E+09	1.386E+03	3.553E+06	1.137E+04	1.395E+03		Mean	3.319E+03	3.093E+03	3.098E+03	3.092E+03	3.094E+03
	Std	6.320E+08	1.089E+02	2.030E+06	9.209E+03	1.311E+02		Std	7.996E+01	1.946E+00	2.439E+00	2.490E+00	1.543E+00
F12	Best	2.253E+05	1.303E+03	1.973E+03	1.303E+03	1.301E+03	F27	Best	3.464E+03	3.100E+03	3.090E+03	2.800E+03	3.100E+03
	Mean	8.389E+07	1.310E+03	9.609E+03	1.317E+03	1.309E+03		Mean	3.851E+03	3.197E+03	3.209E+03	3.159E+03	3.168E+03
	Std	8.428E+07	3.546E+00	6.426E+03	7.651E+00	4.157E+00		Std	1.342E+02	1.403E+02	8.947E+01	1.437E+02	1.257E+02
F13	Best	1.654E+03	1.409E+03	1.441E+03	1.402E+03	1.401E+03	F28	Best	3.419E+03	3.163E+03	3.188E+03	3.166E+03	3.153E+03
	Mean	2.840E+05	1.416E+03	1.596E+03	1.416E+03	1.410E+03		Mean	3.744E+03	3.191E+03	3.227E+03	3.186E+03	3.180E+03
	Std	6.030E+05	4.346E+00	2.724E+02	9.981E+00	6.457E+00		Std	1.483E+02	1.607E+01	2.012E+01	1.631E+01	1.488E+01
F14	Best	1.531E+04	1.501E+03	1.565E+03	1.501E+03	1.501E+03	F29	Best	3.125E+06	3.411E+03	3.646E+04	3.586E+03	3.415E+03
	Mean	2.000E+06	1.503E+03	2.013E+03	1.504E+03	1.502E+03		Mean	7.109E+07	4.526E+04	4.062E+05	1.322E+05	1.140E+05
	Std	4.195E+06	8.736E-01	4.724E+02	1.351E+00	7.093E-01		Std	4.385E+07	2.286E+05	4.074E+05	3.358E+05	3.433E+05
F15	Best	1.926E+03	1.604E+03	1.621E+03	1.602E+03	1.602E+03							
	Mean	2.485E+03	1.618E+03	1.657E+03	1.617E+03	1.609E+03							
	Std	2.084E+02	1.396E+01	3.639E+01	3.157E+01	1.120E+01							

**Table A2:** The statistical results of MSDCS with different strategies for solving CEC2017 (D = 30).

Function	Index	DCS	DCS-PES	DCS-LPSR	DCS-CDM	MSDCS	Function	Index	DCS	DCS-PES	DCS-LPSR	DCS-CDM	MSDCS
F1	Best	6.968E+10	2.270E+02	4.068E+07	3.478E+02	1.279E+02	F16	Best	3.739E+03	1.923E+03	1.984E+03	1.815E+03	1.847E+03
	Mean	8.010E+10	2.605E+03	8.142E+07	3.932E+03	2.443E+03		Mean	5.844E+03	2.098E+03	2.273E+03	2.006E+03	2.055E+03
	Std	2.634E+09	2.095E+03	2.582E+07	3.772E+03	2.807E+03		Std	2.641E+03	1.038E+02	1.194E+02	1.402E+02	1.213E+02
F2	Best	9.466E+04	3.021E+02	8.756E+04	7.698E+02	3.008E+02	F17	Best	9.494E+06	1.830E+03	1.766E+05	2.951E+03	1.843E+03
	Mean	3.768E+05	4.010E+02	1.139E+05	1.498E+03	3.422E+02		Mean	1.955E+08	1.887E+03	1.480E+06	1.669E+04	1.888E+03
	Std	5.610E+05	9.564E+01	1.740E+04	6.535E+02	6.647E+01		Std	1.562E+08	4.374E+01	6.999E+05	1.161E+04	3.026E+01
F3	Best	9.913E+03	4.186E+02	5.456E+02	4.061E+02	4.064E+02	F18	Best	9.253E+08	1.923E+03	2.996E+05	1.951E+03	1.913E+03
	Mean	2.296E+04	4.904E+02	5.803E+02	4.899E+02	4.879E+02		Mean	3.318E+09	1.942E+03	1.311E+06	2.170E+03	1.932E+03
	Std	2.739E+03	2.344E+01	1.761E+01	2.310E+01	2.174E+01		Std	6.716E+08	1.103E+01	9.060E+05	4.597E+02	9.265E+00
F4	Best	1.007E+03	6.126E+02	6.440E+02	5.967E+02	6.084E+02	F19	Best	3.027E+03	2.257E+03	2.481E+03	2.128E+03	2.132E+03
	Mean	1.017E+03	6.452E+02	6.690E+02	6.300E+02	6.266E+02		Mean	3.451E+03	2.511E+03	2.615E+03	2.376E+03	2.375E+03
	Std	1.419E+01	1.360E+01	1.091E+01	1.403E+01	1.037E+01		Std	1.604E+02	1.353E+02	7.827E+01	1.403E+02	1.089E+02
F5	Best	6.882E+02	6.000E+02	6.023E+02	6.000E+02	6.000E+02	F20	Best	2.705E+03	2.437E+03	2.441E+03	2.400E+03	2.402E+03
	Mean	7.103E+02	6.001E+02	6.036E+02	6.001E+02	6.000E+02		Mean	2.849E+03	2.450E+03	2.468E+03	2.428E+03	2.426E+03
	Std	1.028E+01	1.318E-02	7.271E-01	8.160E-02	2.205E-03		Std	5.269E+01	8.612E+00	1.269E+01	1.389E+01	1.119E+01
F6	Best	1.587E+03	8.623E+02	9.010E+02	8.406E+02	8.468E+02	F21	Best	9.019E+03	2.300E+03	2.322E+03	2.300E+03	2.300E+03
	Mean	1.620E+03	8.816E+02	9.275E+02	8.689E+02	8.623E+02		Mean	1.072E+04	2.300E+03	2.328E+03	2.301E+03	2.300E+03
	Std	3.374E+01	1.017E+01	1.375E+01	1.263E+01	9.628E+00		Std	7.906E+02	2.376E-02	3.647E+00	1.280E+00	4.113E-04
F7	Best	1.237E+03	9.322E+02	9.394E+02	8.595E+02	9.080E+02	F22	Best	3.493E+03	2.771E+03	2.778E+03	2.717E+03	2.742E+03
	Mean	1.260E+03	9.491E+02	9.684E+02	9.306E+02	9.286E+02		Mean	3.664E+03	2.796E+03	2.817E+03	2.767E+03	2.770E+03
	Std	1.959E+01	7.717E+00	1.223E+01	1.780E+01	1.019E+01		Std	1.150E+02	1.161E+01	1.327E+01	1.953E+01	1.210E+01
F8	Best	1.574E+04	9.000E+02	9.963E+02	9.001E+02	9.000E+02	F23	Best	3.609E+03	2.928E+03	2.970E+03	2.850E+03	2.852E+03
	Mean	2.080E+04	9.000E+02	1.171E+03	9.070E+02	9.001E+02		Mean	3.962E+03	2.959E+03	2.992E+03	2.917E+03	2.920E+03
	Std	1.933E+03	4.982E-02	1.166E+02	6.199E+00	1.970E-01		Std	1.995E+02	1.334E+01	1.053E+01	3.683E+01	2.926E+01
F9	Best	7.817E+03	6.502E+03	6.390E+03	5.360E+03	5.699E+03	F24	Best	7.213E+03	2.887E+03	2.913E+03	2.887E+03	2.887E+03
	Mean	9.176E+03	7.156E+03	6.929E+03	6.289E+03	6.607E+03		Mean	7.285E+03	2.887E+03	2.942E+03	2.888E+03	2.887E+03
	Std	7.033E+02	3.388E+02	2.581E+02	3.419E+02	2.801E+02		Std	1.365E+02	3.421E-01	2.009E+01	3.119E+00	2.911E-01
F10	Best	8.764E+03	1.112E+03	1.425E+03	1.144E+03	1.109E+03	F25	Best	1.058E+04	4.315E+03	2.936E+03	2.900E+03	2.900E+03
	Mean	2.074E+04	1.160E+03	1.637E+03	1.205E+03	1.142E+03		Mean	1.338E+04	4.918E+03	4.634E+03	4.642E+03	4.440E+03
	Std	6.427E+03	3.859E+01	1.602E+02	4.007E+01	2.644E+01		Std	1.373E+03	1.929E+02	8.457E+02	4.237E+02	4.810E+02
F11	Best	1.101E+10	4.512E+03	6.760E+06	4.065E+04	4.828E+03	F26	Best	3.893E+03	3.186E+03	3.234E+03	3.199E+03	3.175E+03
	Mean	1.799E+10	1.914E+04	2.851E+07	2.323E+05	1.535E+04		Mean	4.811E+03	3.211E+03	3.263E+03	3.212E+03	3.203E+03
	Std	3.544E+09	1.150E+04	1.051E+07	2.533E+05	7.828E+03		Std	3.550E+02	1.067E+01	1.419E+01	7.533E+00	9.145E+00
F12	Best	6.002E+09	1.455E+03	1.352E+06	2.600E+03	1.387E+03	F27	Best	8.047E+03	3.173E+03	3.263E+03	3.206E+03	3.108E+03
	Mean	1.465E+10	1.676E+03	5.353E+06	1.121E+04	1.625E+03		Mean	9.416E+03	3.205E+03	3.309E+03	3.222E+03	3.200E+03
	Std	4.760E+09	1.372E+02	2.878E+06	7.866E+03	1.628E+02		Std	4.558E+02	1.300E+01	2.360E+01	1.533E+01	2.518E+01
F13	Best	3.317E+06	1.444E+03	8.872E+03	1.452E+03	1.439E+03	F28	Best	6.165E+03	3.642E+03	3.955E+03	3.411E+03	3.488E+03
	Mean	1.707E+07	1.473E+03	5.651E+04	1.557E+03	1.461E+03		Mean	1.026E+04	3.825E+03	4.226E+03	3.640E+03	3.657E+03
	Std	1.309E+07	1.117E+01	3.254E+04	5.019E+01	1.223E+01		Std	3.517E+03	1.139E+02	1.255E+02	1.301E+02	9.849E+01
F14	Best	5.795E+08	1.527E+03	1.077E+05	1.654E+03	1.524E+03	F29	Best	3.403E+08	5.623E+03	4.805E+05	8.071E+03	5.380E+03
	Mean	3.195E+09	1.584E+03	7.857E+05	2.042E+03	1.566E+03		Mean	2.340E+09	6.232E+03	1.897E+06	1.351E+04	5.957E+03
	Std	1.372E+09	3.916E+01	4.762E+05	2.299E+02	3.286E+01		Std	9.410E+08	4.660E+02	1.062E+06	4.943E+03	4.161E+02
F15	Best	4.748E+03	2.665E+03	2.722E+03	2.149E+03	2.286E+03							
	Mean	7.231E+03	2.949E+03	3.085E+03	2.759E+03	2.810E+03							
	Std	1.036E+03	1.552E+02	1.883E+02	2.743E+02	1.825E+02							

**Table A3:** The statistical results of MSDCS with different strategies for solving CEC2017 (D = 50).

Function	Index	DCS	DCS-PES	DCS-LPSR	DCS-CDM	MSDCS	Function	Index	DCS	DCS-PES	DCS-LPSR	DCS-CDM	MSDCS
F1	Best	1.349E+11	3.291E+03	8.262E+07	2.836E+03	1.306E+02	F16	Best	1.118E+04	3.134E+03	3.315E+03	2.852E+03	3.068E+03
	Mean	1.351E+11	1.005E+04	2.203E+08	1.344E+04	2.852E+03		Mean	1.183E+05	3.554E+03	3.797E+03	3.274E+03	3.424E+03
	Std	7.063E+08	3.780E+03	9.252E+07	7.381E+03	2.927E+03		Std	6.094E+04	1.978E+02	2.333E+02	2.427E+02	1.657E+02
F2	Best	2.546E+05	2.017E+03	1.664E+05	5.762E+03	2.573E+03	F17	Best	5.761E+07	1.972E+03	1.613E+06	1.219E+04	1.998E+03
	Mean	1.568E+06	5.768E+03	2.370E+05	1.399E+04	5.523E+03		Mean	3.693E+08	2.176E+03	4.114E+06	7.666E+04	2.186E+03
	Std	4.582E+06	2.229E+03	2.333E+04	5.243E+03	1.718E+03		Std	2.065E+08	1.284E+02	1.920E+06	4.934E+04	1.588E+02
F3	Best	4.151E+04	4.319E+02	6.888E+02	5.004E+02	4.764E+02	F18	Best	3.452E+09	1.959E+03	2.781E+05	2.072E+03	1.959E+03
	Mean	5.570E+04	5.518E+02	7.457E+02	5.742E+02	5.478E+02		Mean	7.670E+09	2.031E+03	1.758E+06	1.040E+04	2.014E+03
	Std	3.776E+03	4.499E+01	3.272E+01	3.330E+01	4.058E+01		Std	1.877E+09	3.598E+01	1.054E+06	8.717E+03	4.176E+01
F4	Best	1.316E+03	7.856E+02	8.011E+02	7.367E+02	7.476E+02	F19	Best	4.041E+03	3.156E+03	3.355E+03	2.874E+03	2.920E+03
	Mean	1.353E+03	8.109E+02	8.497E+02	7.831E+02	7.791E+02		Mean	4.711E+03	3.642E+03	3.681E+03	3.488E+03	3.493E+03
	Std	2.671E+01	1.295E+01	1.791E+01	1.618E+01	1.467E+01		Std	3.096E+02	1.690E+02	1.253E+02	2.524E+02	1.750E+02
F5	Best	7.080E+02	6.001E+02	6.040E+02	6.004E+02	6.000E+02	F20	Best	3.140E+03	2.582E+03	2.611E+03	2.542E+03	2.532E+03
	Mean	7.171E+02	6.001E+02	6.054E+02	6.008E+02	6.000E+02		Mean	3.369E+03	2.608E+03	2.644E+03	2.577E+03	2.580E+03
	Std	9.009E+00	2.446E-02	8.224E-01	3.109E-01	7.718E-03		Std	8.072E+01	1.476E+01	1.725E+01	1.814E+01	1.517E+01
F6	Best	2.199E+03	9.939E+02	1.099E+03	1.011E+03	9.816E+02	F21	Best	1.484E+04	2.347E+03	2.409E+03	2.323E+03	2.300E+03
	Mean	2.234E+03	1.057E+03	1.145E+03	1.051E+03	1.032E+03		Mean	1.734E+04	1.312E+04	1.128E+04	1.134E+04	1.215E+04
	Std	4.704E+01	2.125E+01	2.040E+01	1.982E+01	1.580E+01		Std	9.203E+02	2.928E+03	4.429E+03	3.467E+03	3.232E+03
F7	Best	1.592E+03	1.065E+03	1.125E+03	1.019E+03	1.024E+03	F22	Best	4.223E+03	3.001E+03	3.043E+03	2.811E+03	2.938E+03
	Mean	1.623E+03	1.113E+03	1.155E+03	1.078E+03	1.077E+03		Mean	4.719E+03	3.040E+03	3.084E+03	2.989E+03	3.002E+03
	Std	3.300E+01	1.720E+01	1.440E+01	2.050E+01	1.857E+01		Std	2.341E+02	1.833E+01	1.868E+01	4.720E+01	2.159E+01
F8	Best	3.676E+04	9.003E+02	1.378E+03	9.314E+02	9.002E+02	F23	Best	4.523E+03	3.065E+03	3.192E+03	2.974E+03	2.942E+03
	Mean	5.901E+04	9.026E+02	1.857E+03	1.123E+03	9.016E+02		Mean	5.194E+03	3.183E+03	3.248E+03	3.091E+03	3.114E+03
	Std	6.442E+03	2.056E+00	3.592E+02	2.043E+02	1.426E+00		Std	2.586E+02	3.290E+01	1.910E+01	6.805E+01	6.448E+01
F9	Best	1.277E+04	1.139E+04	1.118E+04	1.037E+04	1.021E+04	F24	Best	1.944E+04	2.966E+03	3.137E+03	3.004E+03	2.974E+03
	Mean	1.489E+04	1.234E+04	1.197E+04	1.106E+04	1.145E+04		Mean	1.950E+04	3.033E+03	3.188E+03	3.044E+03	3.030E+03
	Std	9.045E+02	4.225E+02	3.517E+02	2.756E+02	4.800E+02		Std	9.155E+01	2.468E+01	2.386E+01	2.139E+01	2.165E+01
F10	Best	3.620E+04	1.160E+03	2.226E+03	1.187E+03	1.158E+03	F25	Best	1.885E+04	6.070E+03	6.910E+03	4.770E+03	4.542E+03
	Mean	5.580E+04	1.231E+03	2.888E+03	1.285E+03	1.200E+03		Mean	1.966E+04	6.522E+03	7.244E+03	5.917E+03	6.085E+03
	Std	6.723E+03	2.826E+01	5.693E+02	6.231E+01	2.490E+01		Std	2.737E+02	2.185E+02	2.009E+02	5.997E+02	4.009E+02
F11	Best	7.626E+10	1.095E+05	5.381E+07	5.385E+05	1.719E+05	F26	Best	5.741E+03	3.257E+03	3.484E+03	3.254E+03	3.245E+03
	Mean	1.050E+11	8.012E+05	1.260E+08	2.076E+06	5.518E+05		Mean	7.454E+03	3.317E+03	3.575E+03	3.328E+03	3.306E+03
	Std	1.598E+10	6.737E+05	3.494E+07	1.073E+06	3.401E+05		Std	5.898E+02	4.513E+01	6.028E+01	4.486E+01	4.209E+01
F12	Best	3.184E+10	2.633E+03	1.141E+06	2.423E+03	2.439E+03	F27	Best	1.673E+04	3.265E+03	3.356E+03	3.264E+03	3.259E+03
	Mean	4.982E+10	3.406E+03	1.555E+07	8.393E+03	3.547E+03		Mean	1.933E+04	3.312E+03	3.452E+03	3.310E+03	3.287E+03
	Std	1.244E+10	3.761E+02	1.092E+07	4.976E+03	7.059E+02		Std	1.170E+03	3.636E+01	4.421E+01	2.224E+01	2.390E+01
F13	Best	1.749E+07	1.507E+03	1.055E+05	1.668E+03	1.475E+03	F28	Best	1.833E+04	3.796E+03	4.982E+03	3.536E+03	3.756E+03
	Mean	1.336E+08	1.558E+03	4.806E+05	2.987E+03	1.543E+03		Mean	2.987E+05	4.439E+03	5.501E+03	3.931E+03	4.140E+03
	Std	7.684E+07	3.471E+01	1.953E+05	5.288E+03	3.608E+01		Std	2.602E+05	2.629E+02	2.648E+02	2.697E+02	2.266E+02
F14	Best	6.771E+09	1.773E+03	1.001E+06	2.235E+03	1.700E+03	F29	Best	2.875E+09	1.060E+06	2.571E+07	1.187E+06	7.826E+05
	Mean	1.803E+10	1.919E+03	3.428E+06	5.940E+03	1.893E+03		Mean	1.138E+10	1.309E+06	4.482E+07	2.114E+06	9.898E+05
	Std	5.359E+09	8.884E+01	1.365E+06	3.808E+03	1.197E+02		Std	3.338E+09	1.607E+05	1.382E+07	5.076E+05	1.325E+05
F15	Best	9.193E+03	3.854E+03	3.794E+03	2.307E+03	3.413E+03							
	Mean	1.132E+04	4.271E+03	4.607E+03	3.663E+03	4.111E+03							
	Std	1.237E+03	1.898E+02	2.783E+02	6.137E+02	2.339E+02							

**Table A4:** The statistical results of MSDCS with different strategies for solving CEC2017 (D = 100).

Function	Index	DCS	DCS-PES	DCS-LPSR	DCS-CDM	MSDCS	Function	Index	DCS	DCS-PES	DCS-LPSR	DCS-CDM	MSDCS
F1	Best	2.968E+11	9.542E+06	3.979E+08	4.840E+05	1.335E+04	F16	Best	3.008E+06	5.759E+03	6.720E+03	4.274E+03	5.731E+03
	Mean	2.976E+11	1.798E+07	6.953E+08	1.804E+06	2.925E+04		Mean	1.646E+07	6.506E+03	7.237E+03	5.896E+03	6.310E+03
	Std	1.629E+09	4.566E+06	1.846E+08	8.645E+05	1.153E+04		Std	1.285E+07	2.665E+02	2.802E+02	6.713E+02	2.801E+02
F2	Best	3.666E+05	6.134E+04	3.648E+05	5.341E+04	4.792E+04	F17	Best	2.436E+08	3.317E+03	1.675E+06	5.722E+04	5.582E+03
	Mean	1.617E+06	8.012E+04	5.342E+05	8.050E+04	6.645E+04		Mean	6.265E+08	7.778E+03	6.339E+06	2.210E+05	1.579E+04
	Std	3.570E+06	1.422E+04	4.272E+04	1.553E+04	1.140E+04		Std	2.073E+08	2.958E+03	2.686E+06	1.084E+05	6.740E+03
F3	Best	1.291E+05	6.824E+02	9.632E+02	6.468E+02	6.286E+02	F18	Best	2.198E+10	2.474E+03	1.220E+06	2.148E+03	2.162E+03
	Mean	1.596E+05	7.768E+02	1.112E+03	7.252E+02	7.044E+02		Mean	3.444E+10	3.264E+03	7.509E+06	3.828E+03	2.574E+03
	Std	5.875E+03	4.167E+01	6.502E+01	3.641E+01	4.391E+01		Std	5.384E+09	1.399E+03	4.581E+06	2.882E+03	5.819E+02
F4	Best	2.308E+03	1.254E+03	1.291E+03	7.973E+02	1.159E+03	F19	Best	7.480E+03	6.232E+03	6.622E+03	5.851E+03	5.462E+03
	Mean	2.328E+03	1.312E+03	1.353E+03	1.190E+03	1.208E+03		Mean	8.474E+03	6.895E+03	6.998E+03	6.560E+03	6.638E+03
	Std	1.875E+01	2.442E+01	3.130E+01	8.764E+01	2.901E+01		Std	4.279E+02	2.568E+02	2.419E+02	3.087E+02	3.319E+02
F5	Best	7.201E+02	6.016E+02	6.076E+02	6.018E+02	6.001E+02	F20	Best	4.686E+03	3.053E+03	3.105E+03	2.908E+03	2.973E+03
	Mean	7.246E+02	6.021E+02	6.094E+02	6.041E+02	6.002E+02		Mean	5.081E+03	3.138E+03	3.186E+03	3.039E+03	3.037E+03
	Std	3.781E+00	3.045E-01	1.042E+00	1.112E+00	4.938E-02		Std	1.613E+02	3.050E+01	3.650E+01	6.145E+01	2.720E+01
F6	Best	4.234E+03	1.502E+03	1.685E+03	1.542E+03	1.409E+03	F21	Best	3.118E+04	2.328E+03	2.786E+04	2.492E+04	2.661E+04
	Mean	4.315E+03	1.593E+03	1.742E+03	1.640E+03	1.493E+03		Mean	3.396E+04	2.871E+04	2.902E+04	2.695E+04	2.807E+04
	Std	4.467E+01	2.853E+01	2.881E+01	5.471E+01	2.649E+01		Std	1.690E+03	5.012E+03	5.333E+02	6.678E+02	6.535E+02
F7	Best	2.736E+03	1.562E+03	1.579E+03	1.416E+03	1.462E+03	F22	Best	6.374E+03	3.566E+03	3.619E+03	3.080E+03	3.292E+03
	Mean	2.778E+03	1.604E+03	1.643E+03	1.503E+03	1.505E+03		Mean	7.204E+03	3.648E+03	3.674E+03	3.385E+03	3.477E+03
	Std	4.203E+01	2.193E+01	2.974E+01	5.272E+01	2.349E+01		Std	3.676E+02	3.876E+01	2.758E+01	1.402E+02	6.365E+01
F8	Best	1.040E+05	1.061E+03	3.208E+03	3.277E+03	9.376E+02	F23	Best	1.026E+04	3.950E+03	4.031E+03	3.526E+03	3.426E+03
	Mean	1.125E+05	1.220E+03	5.809E+03	7.504E+03	9.904E+02		Mean	1.216E+04	4.058E+03	4.132E+03	3.783E+03	3.840E+03
	Std	6.007E+03	9.719E+01	1.528E+03	3.006E+03	3.938E+01		Std	7.577E+02	4.434E+01	3.839E+01	1.658E+02	1.794E+02
F9	Best	2.928E+04	2.553E+04	2.470E+04	2.310E+04	2.478E+04	F24	Best	3.507E+04	3.364E+03	3.697E+03	3.292E+03	3.211E+03
	Mean	3.147E+04	2.731E+04	2.663E+04	2.450E+04	2.568E+04		Mean	3.519E+04	3.438E+03	3.815E+03	3.395E+03	3.336E+03
	Std	1.261E+03	6.364E+02	5.795E+02	6.272E+02	4.586E+02		Std	2.839E+02	5.333E+01	8.936E+01	5.718E+01	4.470E+01
F10	Best	2.401E+05	2.267E+03	7.506E+04	2.223E+03	1.959E+03	F25	Best	5.893E+04	1.152E+04	1.354E+04	8.153E+03	7.509E+03
	Mean	5.004E+05	2.675E+03	1.291E+05	2.570E+03	2.233E+03		Mean	5.913E+04	1.313E+04	1.434E+04	1.066E+04	1.047E+04
	Std	1.467E+05	2.351E+02	2.627E+04	1.612E+02	1.883E+02		Std	2.846E+02	6.244E+02	4.503E+02	1.577E+03	1.593E+03
F11	Best	2.078E+11	6.007E+06	2.132E+08	3.330E+06	1.267E+06	F26	Best	1.104E+04	3.615E+03	3.695E+03	3.384E+03	3.415E+03
	Mean	2.545E+11	1.898E+07	4.277E+08	1.683E+07	4.716E+06		Mean	1.408E+04	3.730E+03	3.923E+03	3.501E+03	3.510E+03
	Std	1.434E+10	7.757E+06	1.267E+08	8.325E+06	2.522E+06		Std	1.057E+03	7.567E+01	1.151E+02	6.911E+01	4.556E+01
F12	Best	3.576E+10	1.180E+04	7.198E+05	4.303E+03	3.513E+03	F27	Best	3.356E+04	3.494E+03	3.740E+03	3.435E+03	3.402E+03
	Mean	6.282E+10	1.801E+04	7.267E+06	9.456E+03	7.528E+03		Mean	3.368E+04	3.566E+03	3.967E+03	3.507E+03	3.474E+03
	Std	5.774E+09	4.541E+03	8.204E+06	2.986E+03	2.996E+03		Std	1.731E+02	4.227E+01	1.244E+02	3.515E+01	3.775E+01
F13	Best	1.677E+08	1.700E+03	1.414E+06	2.356E+04	1.673E+03	F28	Best	5.127E+05	7.521E+03	8.665E+03	5.003E+03	6.440E+03
	Mean	3.045E+08	1.865E+03	4.117E+06	6.806E+04	1.819E+03		Mean	2.590E+06	8.550E+03	9.721E+03	6.398E+03	7.677E+03
	Std	3.415E+07	9.469E+01	1.494E+06	3.545E+04	9.232E+01		Std	1.542E+06	3.465E+02	4.065E+02	7.818E+02	5.654E+02
F14	Best	2.349E+10	3.190E+03	5.256E+05	2.142E+03	2.098E+03	F29	Best	3.111E+10	5.288E+04	3.136E+06	1.522E+04	1.641E+04
	Mean	3.401E+10	4.217E+03	6.952E+06	3.820E+03	2.574E+03		Mean	5.396E+10	1.101E+05	7.832E+06	5.073E+04	2.463E+04
	Std	4.789E+09	8.085E+02	5.328E+06	2.477E+03	4.791E+02		Std	7.581E+09	4.233E+04	3.430E+06	3.139E+04	5.749E+03
F15	Best	2.123E+04	8.177E+03	8.499E+03	3.794E+03	7.114E+03							
	Mean	3.031E+04	9.068E+03	9.399E+03	6.858E+03	8.320E+03							
	Std	3.732E+03	3.464E+02	3.613E+02	1.515E+03	5.072E+02							

**Table A5:** The statistical results of MSDCS and competition algorithms for solving CEC2017 (D = 10).

Function	Index	MSDCS	DCS	AE	LSHADE	APSM-jSO	RIME	RDGMVO	QIO	MTVSCA	MRFO	EOSMA
F1	Best	1.0000E+02	5.7493E+09	2.0254E+06	3.7831E+05	1.4755E+04	5.3260E+04	2.5642E+02	3.8778E+04	4.7698E+07	9.5523E+04	6.2711E+05
	Mean	1.0000E+02	1.4158E+10	5.9158E+06	3.4316E+06	4.1725E+04	5.5621E+05	1.4843E+04	3.1529E+05	1.6500E+08	3.1513E+05	5.0134E+06
	Std	1.7861E-04	4.4550E+09	2.6245E+06	3.5433E+06	2.2437E+04	4.9964E+05	2.7503E+04	2.2557E+05	7.7902E+07	2.2294E+05	3.3463E+06
	Rank	1	11	9	7	3	6	2	5	10	4	8
F2	Best	3.0000E+02	2.1823E+04	2.4369E+03	3.1469E+02	3.0055E+02	3.1591E+02	3.0000E+02	5.7698E+02	2.7174E+03	3.1261E+02	8.2288E+02
	Mean	3.0000E+02	4.7063E+04	4.9389E+03	7.2511E+02	3.0334E+02	3.7082E+02	3.0012E+02	1.2623E+03	5.1493E+03	4.6712E+02	2.3948E+03
	Std	2.7352E-09	1.7395E+04	1.4506E+03	3.3588E+02	1.9975E+00	5.4313E+01	2.4183E-01	4.6842E+02	1.7953E+03	1.4327E+02	1.3961E+03
	Rank	1	11	9	6	3	4	2	7	10	5	8
F3	Best	4.0007E+02	7.7740E+02	4.0624E+02	4.0446E+02	4.0387E+02	4.0008E+02	4.0037E+02	4.0187E+02	4.1309E+02	4.0200E+02	4.0502E+02
	Mean	4.0066E+02	1.7742E+03	4.0747E+02	4.0708E+02	4.0488E+02	4.1039E+02	4.0814E+02	4.0840E+02	4.2536E+02	4.0801E+02	4.0732E+02
	Std	4.8019E-01	6.8948E+02	4.0923E-01	9.2466E-01	5.4504E-01	1.6528E+01	1.5208E+01	1.1560E+01	8.0864E+00	8.7552E+00	1.4958E+00
	Rank	1	11	5	3	2	9	7	8	10	6	4
F4	Best	5.1030E+02	5.8986E+02	5.2550E+02	5.1768E+02	5.1731E+02	5.0721E+02	5.0500E+02	5.0950E+02	5.3387E+02	5.0804E+02	5.1252E+02
	Mean	5.1618E+02	6.3658E+02	5.3732E+02	5.3883E+02	5.3377E+02	5.1900E+02	5.1911E+02	5.2794E+02	5.4482E+02	5.2538E+02	5.2730E+02
	Std	3.8614E+00	2.2926E+01	6.4713E+00	8.3857E+00	5.8940E+00	6.5260E+00	8.6678E+00	9.2346E+00	4.6029E+00	1.0508E+01	5.7513E+00
	Rank	1	11	8	9	7	2	3	6	10	4	5
F5	Best	6.0000E+02	6.4647E+02	6.0093E+02	6.0161E+02	6.0029E+02	6.0032E+02	6.0007E+02	6.0054E+02	6.0959E+02	6.0037E+02	6.0116E+02
	Mean	6.0000E+02	6.7863E+02	6.0224E+02	6.0380E+02	6.0069E+02	6.0109E+02	6.0025E+02	6.0144E+02	6.1377E+02	6.0158E+02	6.0252E+02
	Std	3.2986E-05	1.3695E+01	6.1605E-01	1.3746E+00	2.2380E-01	6.3389E-01	1.7612E-01	1.0036E+00	2.3962E+00	1.3607E+00	9.9250E-01
	Rank	1	11	7	9	3	4	2	5	10	6	8
F6	Best	7.2033E+02	8.8796E+02	7.3389E+02	7.4647E+02	7.3663E+02	7.1892E+02	7.1378E+02	7.3599E+02	7.4378E+02	7.2989E+02	7.2387E+02
	Mean	7.2806E+02	9.0928E+02	7.4693E+02	7.6061E+02	7.4891E+02	7.3470E+02	7.2823E+02	7.4763E+02	7.7472E+02	7.4960E+02	7.4026E+02
	Std	4.2884E+00	1.5234E+01	5.6237E+00	8.5151E+00	6.0122E+00	9.2727E+00	1.0144E+01	6.1932E+00	9.0861E+00	9.2899E+00	6.1761E+00
	Rank	1	11	5	9	7	3	2	6	10	8	4
F7	Best	8.0794E+02	8.5697E+02	8.1856E+02	8.2438E+02	8.1873E+02	8.0533E+02	8.0697E+02	8.0680E+02	8.2689E+02	8.0618E+02	8.1396E+02
	Mean	8.1653E+02	9.0329E+02	8.3373E+02	8.4130E+02	8.3609E+02	8.1770E+02	8.1901E+02	8.2211E+02	8.4754E+02	8.2092E+02	8.2624E+02
	Std	4.0259E+00	1.3581E+01	7.1353E+00	9.6248E+00	7.1735E+00	6.5434E+00	7.4739E+00	1.0125E+01	7.1703E+00	9.3344E+00	6.3941E+00
	Rank	1	11	7	9	8	2	3	5	10	4	6
F8	Best	9.0000E+02	2.5909E+03	9.0119E+02	9.0247E+02	9.0018E+02	9.0020E+02	9.0000E+02	9.0021E+02	9.4019E+02	9.0039E+02	9.0079E+02
	Mean	9.0000E+02	3.4254E+03	9.0269E+02	9.1259E+02	9.0038E+02	9.0230E+02	9.0515E+02	9.0093E+02	1.0251E+03	9.0545E+02	9.0689E+02
	Std	2.9783E-12	4.5037E+02	1.1132E+00	7.4943E+00	1.3740E-01	3.4621E+00	2.0279E+01	7.3702E-01	5.3334E+01	1.1258E+01	6.6783E+00
	Rank	1	11	5	9	2	4	6	3	10	7	8
F9	Best	1.4374E+03	2.4583E+03	2.1938E+03	2.0536E+03	1.9719E+03	1.0226E+03	1.1260E+03	1.9908E+03	2.1855E+03	1.5052E+03	1.5181E+03
	Mean	1.8821E+03	3.0583E+03	2.6710E+03	2.5458E+03	2.6108E+03	1.5588E+03	1.7127E+03	2.4507E+03	2.5581E+03	2.0555E+03	2.3069E+03
	Std	1.6429E+02	3.2115E+02	2.2077E+02	2.2489E+02	2.2867E+02	2.6641E+02	3.2696E+02	2.2287E+02	2.0811E+02	3.0414E+02	2.6950E+02
	Rank	3	11	10	7	9	1	2	6	8	4	5
F10	Best	1.1029E+03	1.4647E+03	1.1307E+03	1.1090E+03	1.1067E+03	1.1031E+03	1.1021E+03	1.1070E+03	1.1411E+03	1.1105E+03	1.1127E+03
	Mean	1.1044E+03	7.8617E+03	1.1461E+03	1.1204E+03	1.1118E+03	1.1347E+03	1.1140E+03	1.1151E+03	1.1756E+03	1.1200E+03	1.1337E+03
	Std	8.0644E-01	5.2704E+03	1.0143E+01	7.7311E+00	2.4660E+00	6.0100E+01	1.7949E+01	6.2181E+00	1.8774E+01	7.8392E+00	1.5966E+01
	Rank	1	11	9	6	2	8	3	4	10	5	7
F11	Best	1.2005E+03	1.4638E+08	2.1922E+05	8.2101E+03	2.8664E+03	1.2048E+04	1.2600E+04	2.5023E+04	8.1639E+05	1.8912E+04	2.9941E+04
	Mean	1.3946E+03	1.0711E+09	9.8144E+05	1.2265E+05	5.9365E+03	1.2769E+06	6.0515E+05	2.7579E+05	6.7415E+06	1.8522E+05	6.5871E+05
	Std	1.3112E+02	6.3202E+08	5.7889E+05	1.7321E+05	2.7993E+03	1.6782E+06	6.6829E+05	4.7869E+05	4.2237E+06	2.1173E+05	6.0399E+05
	Rank	1	11	8	3	2	9	6	5	10	4	7
F12	Best	1.3008E+03	2.2525E+05	2.9846E+03	1.4033E+03	1.3258E+03	1.8184E+03	1.3242E+03	1.3831E+03	2.9281E+03	1.5709E+03	1.9207E+03
	Mean	1.3089E+03	8.3893E+07	8.5516E+03	1.6971E+03	1.3524E+03	1.0766E+04	9.4812E+03	1.5773E+03	8.6622E+03	8.3282E+03	6.1903E+03
	Std	4.1569E+00	8.4280E+07	2.6780E+03	1.8807E+02	2.3846E+01	8.1255E+03	8.1323E+03	3.4744E+02	5.7888E+03	6.7004E+03	3.2397E+03
	Rank	1	11	7	4	2	10	9	3	8	6	5
F13	Best	1.4010E+03	1.6535E+03	1.4993E+03	1.4220E+03	1.4235E+03	1.4349E+03	1.4091E+03	1.4182E+03	1.4608E+03	1.4530E+03	1.4728E+03
	Mean	1.4095E+03	2.8400E+05	1.6264E+03	1.4410E+03	1.4308E+03	4.3511E+03	6.3744E+03	1.4298E+03	1.5042E+03	1.6244E+03	1.5355E+03
	Std	6.4567E+00	6.0296E+05	1.3233E+02	7.6765E+00	3.1971E+00	3.9295E+03	5.9838E+03	3.5210E+00	3.0758E+01	1.5524E+02	3.4230E+01
	Rank	1	11	8	4	3	9	10	2	5	7	6
F14	Best	1.5005E+03	1.5311E+04	1.7312E+03	1.5124E+03	1.5049E+03	1.5683E+03	1.5030E+03	1.5052E+03	1.6319E+03	1.6031E+03	1.6936E+03
	Mean	1.5016E+03	2.0001E+06	3.2182E+03	1.5439E+03	1.5113E+03	4.2349E+03	6.5258E+03	1.5161E+03	1.9293E+03	2.5458E+03	2.2961E+03
	Std	7.0929E-01	4.1953E+06	1.0759E+03	2.5733E+01	3.4618E+00	4.4649E+03	6.4658E+03	6.1365E+00	2.3096E+02	1.2108E+03	4.4323E+02
	Rank	1	11	8	4	2	9	10	3	5	7	6
F15	Best	1.6020E+03	1.9256E+03	1.6849E+03	1.6245E+03	1.6309E+03	1.6034E+03	1.6007E+03	1.6135E+03	1.6472E+03	1.6027E+03	1.6182E+03
	Mean	1.6089E+03	2.4853E+03	1.7909E+03	1.7087E+03	1.6767E+03	1.7111E+03	1.7449E+03	1.6813E+03	1.8157E+03	1.7629E+03	1.6698E+03
	Std	1.1199E+01	2.0842E+02	7.2196E+01	8.3029E+01	3.9587E+01	1.0266E+02	1.2965E+02	6.2715E+01	7.3752E+01	1.2025E+02	5.9635E+01
	Rank	1	11	9	5	3	6	7	4	10	8	2
F16	Best	1.7079E+03	1.8120E+03	1.7697E+03	1.7433E+03	1.7474E+03	1.7120E+03	1.7009E+03	1.7288E+03	1.7649E+03	1.7301E+03	1.7438E+03
	Mean	1.7274E+03	2.1391E+03	1.8066E+03	1.7763E+03	1.7771E+03	1.7497E+03	1.7353E+03	1.7574E+03	1.8041E+03	1.7539E+03	1.7667E+03
	Std	8.1552E+00	1.5983E+02	2.0363E+01	2.4908E+01	1.3420E+01	3.1434E+01	3.3402E+01	1.8249E+01	2.1915E+01	1.7887E+01	1.2338E+01
	Rank	1	11	10	7	8	3	2	5	9	4	6
F17	Best	1.8005E+03	2.4488E+06	4.6853E+03	1.8901E+03	1.8355E+03	3.0733E+03	6.0183E+03	1.8557E+03	5.0878E+03	3.2995E+03	5.5077E+03
	Mean	1.8051E+03	2.5489E+08	1.4566E+04	2.3704E+03	1.8504E+03	1.9717E+04	2.1238E+04	2.0548E+03	1.8979E+04	1.0591E+04	2.1178E+04
	Std	7.1105E+00	2.2608E+08	7.8372E+03	8.8279E+02	1.1899E+01	1.1309E+04	1.1936E+04	2.4754E+02	1.2195E+04	7.3165E+03	1.6613E+04
	Rank	1	11	6	4	2	8	10	3	7	5	9
F18	Best	1.9004E+03	2.7827E+03	2.0219E+03	1.9071E+03	1.9049E+03	1.9206E+03	1.9066E+03	1.9050E+03	1.9353E+03	1.9761E+03	1.9493E+03
	Mean	1.9013E+03	8.3093E+06	3.5861E+03	1.9167E+03	1.9078E+03	5.9047E+03	6.5940E+03	1.9084E+03	2.2900E+03	3.2191E+03	2.5978E+03
	Std	5.8166E-01	8.1776E+06	1.3430E+03	6.7484E+00	1.4101E+00	4.6855E+03	5.6768E+03	1.7432E+00	4.6198E+02	1.3231E+03	7.1579E+02
	Rank	1	11	8	4	2	9	10	3	5	7	6
F19	Best	2.0075E+03	2.2627E+03	2.0627E+03	2.0329E+03	2.0491E+03	2.0065E+03	2.0005E+03	2.0248E+03	2.0498E+03	2.0131E+03	2.0350E+03
	Mean	2.0205E+03	2.3748E+03	2.1120E+03	2.0604E+03	2.0792E+03	2.0299E+03	2.0215E+03	2.0565E+03	2.1006E+03	2.0597E+03	2.0614E+03
	Std	6.7204E+00	5.9615E+01	2.5586E+01	1.8227E+01	1.3504E+01	1.2538E+01	2.3301E+01	1.6347E+01	2.4887E+01	2.8835E+01	1.4982E+01
	Rank	1	11	10	6	8	3	2	4	9	5	7
F20	Best	2.2000E+03	2.2778E+03	2.2181E+03	2.2048E+03	2.2018E+03	2.2016E+03	2.2000E+03	2.1934E+03	2.2100E+03	2.2006E+03	2.2035E+03
	Mean	2.2637E+03	2.4061E+03	2.3097E+03	2.2778E+03	2.3010E+03	2.2667E+03	2.2822E+03	2.2124E+03	2.2988E+03	2.2480E+03	2.2402E+03
	Std	6.0644E+01	4.7107E+01	4.0100E+01	6.4494E+01	5.8076E+01	6.0394E+01	6.2719E+01	3.2171E+01	5.4048E+01	5.5353E+01	5.1257E+01
	Rank	4	11	10	6	9	5	7	1	8	3	2
F21	Best	2.3000E+03	2.5573E+03	2.3077E+03	2.2605E+03	2.3038E+03	2.2018E+03	2.2226E+03	2.2125E+03	2.2783E+03	2.2232E+03	2.2167E+03
	Mean	2.3004E+03	3.5541E+03	2.3104E+03	2.3090E+03	2.3068E+03	2.2942E+03	2.2988E+03	2.3020E+03	2.3290E+03	2.3027E+03	2.3048E+03
	Std	5.3239E-01	3.9223E+02	1.1781E+00	9.2783E+00	1.3535E+00	3.1612E+01	1.8224E+01	2.2208E+01	1.5785E+01	2.0421E+01	2.1761E+01
	Rank	3	11	9	8	7	1	2	4	10	5	6
F22	Best	2.6044E+03	2.7360E+03	2.6270E+03	2.6147E+03	2.6268E+03	2.6081E+03	2.6090E+03	2.6100E+03	2.6289E+03	2.6136E+03	2.6129E+03
	Mean	2.6135E+03	2.7955E+03	2.6380E+03	2.6317E+03	2.6347E+03	2.6215E+03	2.6235E+03	2.6205E+03	2.6446E+03	2.6288E+03	2.6260E+03
	Std	4.4362E+00	2.4902E+01	5.7527E+00	8.5999E+00	4.6485E+00	6.8253E+00	9.1208E+00	6.1075E+00	7.6926E+00	8.5818E+00	6.7002E+00
	Rank	1	11	9	7	8	3	4	2	10	6	5
F23	Best	2.5000E+03	2.8460E+03	2.7351E+03	2.5157E+03	2.6295E+03	2.5019E+03	2.5001E+03	2.5015E+03	2.5824E+03	2.5015E+03	2.5299E+03
	Mean	2.7215E+03	2.9318E+03	2.7622E+03	2.7487E+03	2.7579E+03	2.6863E+03	2.7069E+03	2.6696E+03	2.7414E+03	2.7379E+03	2.6937E+03
	Std	6.0473E+01	4.9897E+01	7.6980E+00	6.2816E+01	2.8267E+01	1.0637E+02	9.8395E+01	1.1556E+02	6.2861E+01	6.4861E+01	8.7729E+01
	Rank	5	11	10	8	9	2	4	1	7	6	3
F24	Best	2.8977E+03	2.9859E+03	2.9187E+03	2.9011E+03	2.8979E+03	2.8982E+03	2.8978E+03	2.8987E+03	2.9394E+03	2.8980E+03	2.9064E+03
	Mean	2.9270E+03	3.8100E+03	2.9447E+03	2.9345E+03	2.9202E+03	2.9292E+03	2.9307E+03	2.9300E+03	2.9593E+03	2.9216E+03	2.9386E+03
	Std	2.2318E+01	4.0162E+02	9.4120E+00	2.0131E+01	2.3150E+01	2.4955E+01	2.3248E+01	2.1948E+01	6.7199E+00	2.3375E+01	1.6443E+01
	Rank	3	11	9	7	1	4	6	5	10	2	8
F25	Best	2.9000E+03	3.8446E+03	2.9162E+03	2.9035E+03	2.9004E+03	2.6150E+03	2.8006E+03	2.9017E+03	2.9509E+03	2.8182E+03	2.7594E+03
	Mean	2.9000E+03	4.6015E+03	2.9356E+03	2.9222E+03	2.9009E+03	2.9151E+03	2.9616E+03	2.9163E+03	3.0281E+03	2.9189E+03	2.9265E+03
	Std	2.9802E-07	3.3601E+02	1.1421E+01	1.7723E+01	3.3107E-01	7.4756E+01	1.5665E+02	1.6192E+01	4.0095E+01	5.5160E+01	3.7675E+01
	Rank	1	11	8	6	2	3	9	4	10	5	7
F26	Best	3.0900E+03	3.1681E+03	3.0934E+03	3.0900E+03	3.0891E+03	3.0897E+03	3.0737E+03	3.0934E+03	3.1041E+03	3.0935E+03	3.0930E+03
	Mean	3.0941E+03	3.3186E+03	3.0983E+03	3.0934E+03	3.0910E+03	3.0958E+03	3.0924E+03	3.0999E+03	3.1093E+03	3.1041E+03	3.0971E+03
	Std	1.5433E+00	7.9957E+01	1.5598E+00	2.1453E+00	1.5088E+00	3.5479E+00	2.8838E+01	3.3901E+00	2.9186E+00	1.1693E+01	1.6954E+00
	Rank	4	11	7	3	1	5	2	8	10	9	6
F27	Best	3.1000E+03	3.4636E+03	3.1689E+03	3.1197E+03	3.1019E+03	3.1037E+03	3.1001E+03	3.1027E+03	3.2034E+03	3.1043E+03	3.1352E+03
	Mean	3.1681E+03	3.8514E+03	3.2676E+03	3.2111E+03	3.2324E+03	3.2773E+03	3.2312E+03	3.2159E+03	3.2533E+03	3.2690E+03	3.1973E+03
	Std	1.2567E+02	1.3415E+02	7.7804E+01	9.0134E+01	1.3345E+02	1.1233E+02	6.1484E+01	1.0604E+02	3.2703E+01	1.3869E+02	5.8523E+01
	Rank	1	11	8	3	6	10	5	4	7	9	2
F28	Best	3.1525E+03	3.4186E+03	3.2074E+03	3.1814E+03	3.1815E+03	3.1505E+03	3.1366E+03	3.1721E+03	3.1872E+03	3.1644E+03	3.1693E+03
	Mean	3.1802E+03	3.7442E+03	3.2549E+03	3.2263E+03	3.2126E+03	3.1984E+03	3.2084E+03	3.2209E+03	3.2644E+03	3.2193E+03	3.2161E+03
	Std	1.4881E+01	1.4833E+02	2.7033E+01	3.1315E+01	1.9708E+01	4.4018E+01	4.9649E+01	3.0714E+01	3.6303E+01	3.8406E+01	2.6174E+01
	Rank	1	11	9	8	4	2	3	7	10	6	5
F29	Best	3.4150E+03	3.1248E+06	4.0335E+05	5.3272E+03	3.6342E+03	4.4292E+03	3.8858E+03	2.2907E+04	3.9652E+04	7.2975E+03	6.1996E+03
	Mean	1.1400E+05	7.1092E+07	1.3283E+06	2.3745E+05	1.0364E+05	3.9717E+05	2.5083E+04	4.9143E+05	1.2247E+06	7.1444E+05	2.8318E+05
	Std	3.4330E+05	4.3848E+07	8.4507E+05	4.8294E+05	3.0635E+05	5.7051E+05	3.1032E+04	8.8873E+05	1.1072E+06	9.9113E+05	5.7114E+05
	Rank	3	11	10	4	2	6	1	7	9	8	5

**Table A6:** The statistical results of MSDCS and competition algorithms for solving CEC2017 (D = 30).

Function	Index	MSDCS	DCS	AE	LSHADE	APSM-jSO	RIME	RDGMVO	QIO	MTVSCA	MRFO	EOSMA
F1	Best	1.2789E+02	6.9678E+10	6.4842E+07	9.6229E+06	7.0730E+04	1.0115E+07	2.2982E+03	2.5572E+07	1.7446E+09	2.7199E+07	9.1788E+07
	Mean	2.4428E+03	8.0101E+10	1.2490E+08	2.9292E+07	1.2570E+05	2.5748E+07	1.0190E+05	5.6322E+07	3.4245E+09	5.6907E+07	4.0150E+08
	Std	2.8072E+03	2.6339E+09	3.9593E+07	1.3945E+07	4.6680E+04	1.1722E+07	1.5103E+05	1.7546E+07	9.0780E+08	1.9774E+07	1.2956E+08
	Rank	1	11	8	5	3	4	2	6	10	7	9
F2	Best	3.0078E+02	9.4664E+04	5.2191E+04	8.0755E+03	6.3748E+02	1.4127E+04	3.2131E+03	4.5889E+04	4.7636E+04	2.0795E+04	3.6754E+04
	Mean	3.4215E+02	3.7681E+05	7.2093E+04	1.7769E+04	1.6346E+03	2.9966E+04	7.9464E+03	6.0370E+04	6.5992E+04	3.1407E+04	5.7956E+04
	Std	6.6465E+01	5.6101E+05	8.9338E+03	5.1024E+03	8.4654E+02	1.0495E+04	2.6125E+03	1.1281E+04	9.5735E+03	5.2953E+03	1.3805E+04
	Rank	1	11	10	4	2	5	3	8	9	6	7
F3	Best	4.0645E+02	9.9125E+03	5.4047E+02	4.9758E+02	4.8674E+02	4.9797E+02	4.1996E+02	4.9081E+02	7.9115E+02	4.8470E+02	5.4189E+02
	Mean	4.8789E+02	2.2956E+04	5.6575E+02	5.2247E+02	4.9631E+02	5.3192E+02	4.6135E+02	5.5831E+02	9.9079E+02	5.5406E+02	5.8642E+02
	Std	2.1736E+01	2.7394E+03	1.8881E+01	1.2807E+01	1.1765E+01	2.7109E+01	3.3667E+01	3.5079E+01	1.4430E+02	3.4094E+01	2.8049E+01
	Rank	2	11	8	4	3	5	1	7	10	6	9
F4	Best	6.0835E+02	1.0066E+03	6.5531E+02	6.4337E+02	6.6181E+02	5.5826E+02	5.6377E+02	6.1066E+02	7.0609E+02	5.8767E+02	6.2099E+02
	Mean	6.2659E+02	1.0167E+03	7.0006E+02	6.7944E+02	6.9274E+02	5.8825E+02	6.1293E+02	6.6718E+02	7.4485E+02	6.5762E+02	6.5512E+02
	Std	1.0369E+01	1.4189E+01	1.5449E+01	1.6828E+01	1.2268E+01	1.5402E+01	2.7901E+01	3.2730E+01	1.5249E+01	3.8743E+01	1.7986E+01
	Rank	3	11	9	7	8	1	2	6	10	5	4
F5	Best	6.0000E+02	6.8815E+02	6.0517E+02	6.0371E+02	6.0054E+02	6.0299E+02	6.0058E+02	6.0529E+02	6.2645E+02	6.0634E+02	6.0579E+02
	Mean	6.0000E+02	7.1028E+02	6.0717E+02	6.0736E+02	6.0084E+02	6.0855E+02	6.0351E+02	6.1190E+02	6.3074E+02	6.2410E+02	6.1024E+02
	Std	2.2047E-03	1.0282E+01	1.3808E+00	2.3914E+00	1.8008E-01	3.1862E+00	2.6960E+00	3.2483E+00	3.2977E+00	1.4465E+01	2.6056E+00
	Rank	1	11	4	5	2	6	3	8	10	9	7
F6	Best	8.4684E+02	1.5872E+03	8.9452E+02	8.8854E+02	8.9741E+02	8.0976E+02	7.8756E+02	8.9706E+02	1.0035E+03	8.6640E+02	8.7118E+02
	Mean	8.6226E+02	1.6200E+03	9.4096E+02	9.2144E+02	9.2160E+02	8.7769E+02	8.5849E+02	9.4967E+02	1.0557E+03	9.9026E+02	9.1035E+02
	Std	9.6278E+00	3.3744E+01	1.7434E+01	1.7586E+01	8.6095E+00	2.9992E+01	3.8109E+01	2.0903E+01	2.9602E+01	5.5529E+01	2.1628E+01
	Rank	2	11	7	5	6	3	1	8	10	9	4
F7	Best	9.0802E+02	1.2371E+03	9.5693E+02	9.2519E+02	9.5760E+02	8.5711E+02	8.6071E+02	8.7822E+02	9.9985E+02	8.8133E+02	9.0705E+02
	Mean	9.2857E+02	1.2602E+03	9.9478E+02	9.7446E+02	9.8561E+02	8.8969E+02	8.9980E+02	9.3115E+02	1.0358E+03	9.3048E+02	9.4591E+02
	Std	1.0187E+01	1.9590E+01	1.5720E+01	2.2990E+01	1.1417E+01	2.3824E+01	2.8064E+01	3.2196E+01	1.6353E+01	2.5559E+01	1.8321E+01
	Rank	3	11	9	7	8	1	2	5	10	4	6
F8	Best	9.0000E+02	1.5743E+04	9.5161E+02	9.5029E+02	9.0077E+02	1.0303E+03	1.3332E+03	1.0111E+03	2.0751E+03	1.7020E+03	1.0216E+03
	Mean	9.0006E+02	2.0803E+04	1.0526E+03	1.0566E+03	9.0186E+02	1.7186E+03	3.3263E+03	1.2360E+03	3.1588E+03	3.8599E+03	1.3133E+03
	Std	1.9701E-01	1.9327E+03	6.2455E+01	7.2868E+01	1.3727E+00	5.2236E+02	1.3637E+03	2.1169E+02	5.9128E+02	1.3182E+03	2.1910E+02
	Rank	1	11	3	4	2	7	9	5	8	10	6
F9	Best	5.6989E+03	7.8165E+03	8.0205E+03	5.9157E+03	7.9234E+03	3.1085E+03	3.1814E+03	7.5132E+03	7.8360E+03	4.2946E+03	7.0040E+03
	Mean	6.6065E+03	9.1763E+03	8.7444E+03	8.0210E+03	8.6795E+03	4.5889E+03	4.0954E+03	8.5351E+03	8.6381E+03	6.3286E+03	7.8182E+03
	Std	2.8013E+02	7.0332E+02	3.1874E+02	6.6958E+02	2.7523E+02	7.0624E+02	6.3033E+02	3.3318E+02	2.7112E+02	9.6997E+02	3.8077E+02
	Rank	4	11	10	6	9	2	1	7	8	3	5
F10	Best	1.1094E+03	8.7639E+03	1.6348E+03	1.2109E+03	1.1784E+03	1.2140E+03	1.1416E+03	1.2816E+03	1.6305E+03	1.3427E+03	1.4262E+03
	Mean	1.1424E+03	2.0738E+04	1.8026E+03	1.3096E+03	1.2175E+03	1.3523E+03	1.1845E+03	1.3396E+03	1.9944E+03	1.4310E+03	1.6081E+03
	Std	2.6436E+01	6.4274E+03	1.1884E+02	4.6648E+01	3.0352E+01	9.1755E+01	3.2090E+01	3.2309E+01	2.3648E+02	6.7219E+01	1.0736E+02
	Rank	1	11	9	4	3	6	2	5	10	7	8
F11	Best	4.8283E+03	1.1012E+10	4.7083E+06	3.6529E+05	3.2532E+04	2.6023E+06	5.3799E+05	1.1219E+06	1.0275E+08	3.3893E+05	3.1825E+06
	Mean	1.5346E+04	1.7992E+10	8.6813E+06	2.9070E+06	1.8758E+05	1.9415E+07	4.5416E+06	5.1306E+06	2.2206E+08	4.9188E+06	1.4806E+07
	Std	7.8276E+03	3.5436E+09	2.6514E+06	3.6250E+06	1.5335E+05	1.5158E+07	3.9333E+06	3.0300E+06	7.5342E+07	4.1107E+06	1.1167E+07
	Rank	1	11	7	3	2	9	4	6	10	5	8
F12	Best	1.3874E+03	6.0015E+09	1.1837E+05	3.4413E+04	7.8270E+03	7.3724E+04	2.6081E+03	1.4751E+04	1.2668E+07	1.1394E+04	6.6095E+04
	Mean	1.6245E+03	1.4654E+10	2.9136E+05	9.5555E+04	1.5153E+04	2.4881E+05	2.1622E+04	3.3656E+04	3.2745E+07	3.6475E+04	2.6494E+05
	Std	1.6281E+02	4.7599E+09	9.7410E+04	5.1504E+04	6.9316E+03	2.4078E+05	2.5195E+04	1.8523E+04	1.2221E+07	4.3226E+04	2.0220E+05
	Rank	1	11	9	6	2	7	3	4	10	5	8
F13	Best	1.4389E+03	3.3165E+06	1.3025E+04	1.5570E+03	1.4851E+03	5.5984E+03	2.5114E+03	5.3237E+03	1.9818E+04	3.3197E+03	4.7445E+03
	Mean	1.4605E+03	1.7072E+07	4.6612E+04	1.6790E+03	1.5228E+03	5.9309E+04	4.4655E+04	2.3843E+04	4.8016E+04	3.8729E+04	2.4547E+04
	Std	1.2228E+01	1.3086E+07	2.1699E+04	9.5858E+01	1.3373E+01	4.8589E+04	3.4230E+04	1.9080E+04	2.3984E+04	3.2537E+04	1.8724E+04
	Rank	1	11	8	3	2	10	7	4	9	6	5
F14	Best	1.5245E+03	5.7946E+08	2.4078E+04	6.5196E+03	1.9318E+03	1.6141E+04	1.6334E+03	4.2687E+03	4.9801E+05	2.2119E+03	1.3672E+04
	Mean	1.5660E+03	3.1947E+09	4.3102E+04	2.0277E+04	2.2963E+03	6.9537E+04	8.6588E+03	9.7852E+03	2.5862E+06	1.0344E+04	5.2308E+04
	Std	3.2864E+01	1.3721E+09	1.1856E+04	1.3156E+04	2.1917E+02	4.1652E+04	8.9135E+03	4.9710E+03	1.5826E+06	1.1114E+04	3.0419E+04
	Rank	1	11	7	6	2	9	3	4	10	5	8
F15	Best	2.2858E+03	4.7477E+03	2.7115E+03	2.6878E+03	2.8858E+03	1.8709E+03	2.1759E+03	2.1550E+03	3.3343E+03	2.4338E+03	2.2428E+03
	Mean	2.8099E+03	7.2309E+03	3.4056E+03	3.2775E+03	3.2492E+03	2.5671E+03	2.5603E+03	2.9042E+03	3.6385E+03	2.8167E+03	2.7722E+03
	Std	1.8253E+02	1.0356E+03	1.9267E+02	2.3919E+02	1.7570E+02	2.8530E+02	2.3818E+02	4.2004E+02	1.6902E+02	2.8056E+02	3.1813E+02
	Rank	4	11	9	8	7	2	1	6	10	5	3
F16	Best	1.8474E+03	3.7393E+03	2.1346E+03	1.9849E+03	1.9885E+03	1.7723E+03	1.7682E+03	1.8012E+03	2.0818E+03	1.8130E+03	1.8357E+03
	Mean	2.0549E+03	5.8444E+03	2.4139E+03	2.2427E+03	2.2746E+03	2.1119E+03	2.0794E+03	1.9991E+03	2.4667E+03	2.0926E+03	2.0163E+03
	Std	1.2127E+02	2.6407E+03	1.1498E+02	1.3594E+02	1.1767E+02	1.7694E+02	1.7424E+02	1.7936E+02	1.5564E+02	1.7923E+02	1.4197E+02
	Rank	3	11	9	7	8	6	4	1	10	5	2
F17	Best	1.8427E+03	9.4938E+06	1.6473E+05	5.9353E+03	2.1495E+03	1.5659E+05	8.7134E+04	1.1633E+05	3.6184E+05	6.6498E+04	1.4903E+05
	Mean	1.8879E+03	1.9552E+08	6.9501E+05	3.6252E+04	2.7279E+03	1.5461E+06	1.0152E+06	5.6277E+05	1.2128E+06	5.9050E+05	6.8045E+05
	Std	3.0259E+01	1.5622E+08	3.5416E+05	1.7018E+04	6.4050E+02	1.4528E+06	1.0071E+06	3.8426E+05	6.9813E+05	7.6539E+05	5.3510E+05
	Rank	1	11	7	3	2	10	8	4	9	5	6
F18	Best	1.9129E+03	9.2534E+08	5.0720E+04	2.7846E+03	2.0634E+03	2.6605E+04	1.9848E+03	2.7186E+03	5.9694E+05	2.1459E+03	9.8101E+03
	Mean	1.9319E+03	3.3185E+09	1.3818E+05	1.1072E+04	2.1566E+03	2.5734E+05	1.2363E+04	9.9789E+03	3.6871E+06	1.1886E+04	1.3711E+05
	Std	9.2647E+00	6.7163E+08	6.0497E+04	6.5001E+03	6.1788E+01	2.3388E+05	1.6566E+04	7.2472E+03	2.3170E+06	1.3210E+04	1.4307E+05
	Rank	1	11	8	4	2	9	6	3	10	5	7
F19	Best	2.1320E+03	3.0274E+03	2.6063E+03	2.3259E+03	2.5950E+03	2.0985E+03	2.0560E+03	2.1806E+03	2.6763E+03	2.2454E+03	2.1971E+03
	Mean	2.3746E+03	3.4508E+03	2.7865E+03	2.6830E+03	2.7747E+03	2.4425E+03	2.4687E+03	2.4758E+03	2.8590E+03	2.4728E+03	2.4505E+03
	Std	1.0893E+02	1.6038E+02	1.0155E+02	2.0074E+02	9.9529E+01	1.6048E+02	1.7922E+02	1.5605E+02	1.1333E+02	1.6826E+02	1.1333E+02
	Rank	1	11	9	7	8	2	4	6	10	5	3
F20	Best	2.4020E+03	2.7045E+03	2.4590E+03	2.4144E+03	2.4569E+03	2.3642E+03	2.3531E+03	2.3782E+03	2.4897E+03	2.2241E+03	2.4093E+03
	Mean	2.4256E+03	2.8492E+03	2.4924E+03	2.4719E+03	2.4818E+03	2.4016E+03	2.4075E+03	2.4333E+03	2.5342E+03	2.4277E+03	2.4434E+03
	Std	1.1193E+01	5.2695E+01	1.1772E+01	2.2443E+01	1.2215E+01	2.6143E+01	3.2462E+01	3.4799E+01	1.4527E+01	4.2532E+01	1.8105E+01
	Rank	3	11	9	7	8	1	2	5	10	4	6
F21	Best	2.3000E+03	9.0188E+03	2.3432E+03	2.3302E+03	2.3042E+03	2.3219E+03	2.3006E+03	2.3318E+03	2.6608E+03	2.3345E+03	2.3801E+03
	Mean	2.3000E+03	1.0720E+04	2.3679E+03	2.3549E+03	2.3095E+03	3.7835E+03	3.0755E+03	2.3452E+03	3.6784E+03	2.3792E+03	2.4453E+03
	Std	4.1125E-04	7.9063E+02	1.5171E+01	1.7305E+01	2.2206E+00	1.9636E+03	1.3395E+03	6.6221E+00	1.4148E+03	1.8731E+02	9.0232E+01
	Rank	1	11	5	4	2	10	8	3	9	6	7
F22	Best	2.7417E+03	3.4926E+03	2.8187E+03	2.8029E+03	2.8119E+03	2.7142E+03	2.7118E+03	2.7653E+03	2.8498E+03	2.7530E+03	2.7389E+03
	Mean	2.7700E+03	3.6640E+03	2.8438E+03	2.8318E+03	2.8399E+03	2.7485E+03	2.7688E+03	2.8409E+03	2.9068E+03	2.8231E+03	2.7939E+03
	Std	1.2097E+01	1.1501E+02	1.5672E+01	1.4353E+01	1.1327E+01	2.6539E+01	3.0882E+01	3.3583E+01	2.0937E+01	3.6225E+01	2.2704E+01
	Rank	3	11	9	6	7	1	2	8	10	5	4
F23	Best	2.8517E+03	3.6088E+03	2.9730E+03	2.9124E+03	2.9774E+03	2.8758E+03	2.8870E+03	2.9166E+03	3.0240E+03	2.9340E+03	2.9259E+03
	Mean	2.9201E+03	3.9615E+03	3.0106E+03	2.9942E+03	3.0040E+03	2.9097E+03	2.9446E+03	2.9854E+03	3.0689E+03	3.0063E+03	2.9601E+03
	Std	2.9263E+01	1.9954E+02	1.9380E+01	2.4202E+01	1.1858E+01	2.1289E+01	3.2188E+01	3.5366E+01	1.7459E+01	5.3729E+01	1.8173E+01
	Rank	2	11	9	6	7	1	3	5	10	8	4
F24	Best	2.8868E+03	7.2135E+03	2.9294E+03	2.8927E+03	2.8872E+03	2.8973E+03	2.8775E+03	2.9045E+03	3.0675E+03	2.9079E+03	2.9302E+03
	Mean	2.8871E+03	7.2849E+03	2.9639E+03	2.9089E+03	2.8878E+03	2.9313E+03	2.8907E+03	2.9475E+03	3.1837E+03	2.9483E+03	2.9806E+03
	Std	2.9114E-01	1.3651E+02	1.5995E+01	1.1790E+01	7.4233E-01	2.5773E+01	1.4471E+01	2.8692E+01	6.2297E+01	1.9487E+01	2.1447E+01
	Rank	1	11	8	4	2	5	3	6	10	7	9
F25	Best	2.9000E+03	1.0578E+04	5.3542E+03	4.9122E+03	5.0895E+03	4.1261E+03	2.8030E+03	2.9820E+03	4.5671E+03	3.0838E+03	3.5112E+03
	Mean	4.4405E+03	1.3379E+04	5.6667E+03	5.3164E+03	5.3737E+03	4.7998E+03	4.5571E+03	4.8837E+03	6.2411E+03	5.0061E+03	4.9088E+03
	Std	4.8095E+02	1.3730E+03	1.3579E+02	1.9655E+02	1.1541E+02	3.0929E+02	9.4378E+02	9.2771E+02	4.4505E+02	1.0979E+03	4.9019E+02
	Rank	1	11	9	7	8	3	2	4	10	6	5
F26	Best	3.1752E+03	3.8929E+03	3.2496E+03	3.2142E+03	3.2043E+03	3.2099E+03	3.1655E+03	3.2622E+03	3.3259E+03	3.2599E+03	3.2441E+03
	Mean	3.2030E+03	4.8110E+03	3.2713E+03	3.2376E+03	3.2220E+03	3.2314E+03	3.1877E+03	3.3004E+03	3.3761E+03	3.3063E+03	3.2659E+03
	Std	9.1451E+00	3.5498E+02	1.2823E+01	1.2713E+01	7.8312E+00	1.2674E+01	1.1917E+01	2.4611E+01	2.4826E+01	2.7096E+01	1.3713E+01
	Rank	2	11	7	5	3	4	1	8	10	9	6
F27	Best	3.1082E+03	8.0469E+03	3.3076E+03	3.2420E+03	3.2099E+03	3.2431E+03	3.2010E+03	3.2654E+03	3.4919E+03	3.2393E+03	3.2801E+03
	Mean	3.1997E+03	9.4157E+03	3.3401E+03	3.2702E+03	3.2339E+03	3.3164E+03	3.2433E+03	3.3171E+03	3.6307E+03	3.3236E+03	3.3585E+03
	Std	2.5184E+01	4.5578E+02	1.5935E+01	1.7252E+01	1.4000E+01	4.6283E+01	3.1649E+01	2.2777E+01	8.2890E+01	2.9490E+01	3.0828E+01
	Rank	1	11	8	4	2	5	3	6	10	7	9
F28	Best	3.4879E+03	6.1648E+03	3.9332E+03	3.7051E+03	3.8226E+03	3.5757E+03	3.5029E+03	3.6068E+03	4.1640E+03	3.5471E+03	3.6585E+03
	Mean	3.6567E+03	1.0257E+04	4.2599E+03	4.0688E+03	4.0290E+03	3.8564E+03	3.7994E+03	3.8383E+03	4.5583E+03	3.9188E+03	3.8653E+03
	Std	9.8493E+01	3.5170E+03	1.3816E+02	1.9884E+02	1.2625E+02	2.2218E+02	1.3203E+02	1.6027E+02	1.7583E+02	2.0898E+02	1.3594E+02
	Rank	1	11	9	8	7	4	2	3	10	6	5
F29	Best	5.3796E+03	3.4033E+08	3.6473E+05	3.7088E+04	8.7309E+03	2.2549E+05	9.3382E+03	5.5908E+04	2.2886E+06	2.9447E+04	2.5746E+05
	Mean	5.9567E+03	2.3402E+09	1.0855E+06	1.7177E+05	1.5522E+04	1.9855E+06	9.5918E+04	2.5124E+05	9.6583E+06	1.6342E+05	1.4191E+06
	Std	4.1611E+02	9.4098E+08	4.6725E+05	1.2804E+05	5.6107E+03	1.6168E+06	1.4589E+05	1.4015E+05	3.7327E+06	1.1588E+05	9.9749E+05
	Rank	1	11	7	5	2	9	3	6	10	4	8

**Table A7:** The statistical results of MSDCS and competition algorithms for solving CEC2017 (D = 50).

Function	Index	MSDCS	DCS	AE	LSHADE	APSM-jSO	RIME	RDGMVO	QIO	MTVSCA	MRFO	EOSMA
F1	Best	1.3060E+02	1.3487E+11	4.2215E+08	2.8919E+07	3.7483E+05	7.6035E+07	3.8773E+04	2.0545E+08	7.5938E+09	4.3024E+08	1.1567E+09
	Mean	2.8517E+03	1.3512E+11	6.8873E+08	8.6544E+07	7.8669E+05	1.6704E+08	3.4845E+05	4.0192E+08	1.2956E+10	9.4278E+08	2.7588E+09
	Std	2.9271E+03	7.0630E+08	1.6210E+08	4.1883E+07	2.6999E+05	6.9993E+07	4.4701E+05	1.0596E+08	2.9357E+09	4.4807E+08	9.3678E+08
	Rank	1	11	7	4	3	5	2	6	10	8	9
F2	Best	2.5734E+03	2.5461E+05	1.2998E+05	3.6202E+04	6.9610E+03	5.7080E+04	2.8103E+04	1.0508E+05	1.2495E+05	8.5142E+04	8.8133E+04
	Mean	5.5231E+03	1.5675E+06	1.6013E+05	5.4716E+04	1.2646E+04	1.0325E+05	4.8394E+04	1.4005E+05	1.5326E+05	1.3356E+05	1.3827E+05
	Std	1.7185E+03	4.5821E+06	1.3530E+04	1.2483E+04	3.5464E+03	1.8245E+04	8.3873E+03	1.8679E+04	2.0486E+04	2.2917E+04	1.9125E+04
	Rank	1	11	10	4	2	5	3	8	9	6	7
F3	Best	4.7642E+02	4.1506E+04	7.1041E+02	5.8647E+02	4.9276E+02	5.9302E+02	4.2954E+02	6.7027E+02	1.5596E+03	6.5812E+02	7.6102E+02
	Mean	5.4778E+02	5.5700E+04	7.8001E+02	6.5073E+02	5.7022E+02	6.8437E+02	5.1726E+02	7.7413E+02	2.2954E+03	8.7480E+02	9.0700E+02
	Std	4.0575E+01	3.7760E+03	3.5786E+01	2.2762E+01	4.4658E+01	5.5145E+01	5.3526E+01	5.2179E+01	4.8432E+02	1.2401E+02	1.0004E+02
	Rank	2	11	7	4	3	5	1	6	10	8	9
F4	Best	7.4758E+02	1.3156E+03	8.4938E+02	7.4976E+02	8.3081E+02	6.3793E+02	6.4486E+02	7.3284E+02	8.9207E+02	7.4469E+02	7.4524E+02
	Mean	7.7911E+02	1.3527E+03	8.9848E+02	8.1565E+02	8.5801E+02	7.1134E+02	7.2982E+02	8.0836E+02	9.5744E+02	8.2743E+02	8.0193E+02
	Std	1.4673E+01	2.6714E+01	2.0168E+01	2.8152E+01	1.2455E+01	4.4418E+01	4.2303E+01	5.7194E+01	2.3723E+01	4.2789E+01	2.8019E+01
	Rank	3	11	9	6	8	1	2	5	10	7	4
F5	Best	6.0001E+02	7.0803E+02	6.0733E+02	6.0560E+02	6.0072E+02	6.0849E+02	6.0301E+02	6.1272E+02	6.2690E+02	6.2569E+02	6.0992E+02
	Mean	6.0002E+02	7.1706E+02	6.1037E+02	6.0871E+02	6.0109E+02	6.1760E+02	6.1188E+02	6.2015E+02	6.4226E+02	6.4499E+02	6.1747E+02
	Std	7.7178E-03	9.0086E+00	1.6222E+00	1.8354E+00	2.6473E-01	4.7498E+00	6.5775E+00	4.8722E+00	4.9215E+00	1.1735E+01	4.0195E+00
	Rank	1	11	4	3	2	7	5	8	9	10	6
F6	Best	9.8160E+02	2.1987E+03	1.1327E+03	1.0598E+03	1.0743E+03	1.0257E+03	8.9539E+02	1.1033E+03	1.2955E+03	1.0907E+03	1.0715E+03
	Mean	1.0323E+03	2.2338E+03	1.1818E+03	1.0908E+03	1.1087E+03	1.0981E+03	1.0003E+03	1.1924E+03	1.3870E+03	1.3078E+03	1.1408E+03
	Std	1.5797E+01	4.7037E+01	1.9328E+01	1.8159E+01	1.3347E+01	4.1775E+01	6.0251E+01	4.2940E+01	4.7642E+01	1.0490E+02	4.0208E+01
	Rank	2	11	7	3	5	4	1	8	10	9	6
F7	Best	1.0240E+03	1.5916E+03	1.1554E+03	1.0335E+03	1.1062E+03	9.3894E+02	9.5628E+02	9.6794E+02	1.1992E+03	1.0283E+03	1.0427E+03
	Mean	1.0772E+03	1.6229E+03	1.1960E+03	1.1138E+03	1.1584E+03	1.0202E+03	1.0290E+03	1.0930E+03	1.2641E+03	1.1307E+03	1.1166E+03
	Std	1.8567E+01	3.3004E+01	1.6907E+01	3.1735E+01	1.7242E+01	3.6137E+01	4.6512E+01	5.3815E+01	2.6081E+01	4.6686E+01	2.8551E+01
	Rank	3	11	9	5	8	1	2	4	10	7	6
F8	Best	9.0018E+02	3.6764E+04	1.1867E+03	1.1759E+03	9.0285E+02	1.7813E+03	4.3217E+03	1.6739E+03	6.7774E+03	9.0592E+03	2.0770E+03
	Mean	9.0164E+02	5.9013E+04	1.7824E+03	1.3990E+03	9.0771E+02	5.7289E+03	8.8040E+03	4.1463E+03	1.0221E+04	1.9846E+04	4.0539E+03
	Std	1.4258E+00	6.4422E+03	2.8044E+02	1.4490E+02	4.1750E+00	2.6091E+03	3.2487E+03	1.2682E+03	1.9007E+03	5.7257E+03	1.1967E+03
	Rank	1	11	4	3	2	7	8	6	9	10	5
F9	Best	1.0209E+04	1.2768E+04	1.4040E+04	1.2129E+04	1.3794E+04	6.9993E+03	5.3035E+03	1.3329E+04	1.3850E+04	7.6160E+03	1.1690E+04
	Mean	1.1449E+04	1.4889E+04	1.4973E+04	1.3932E+04	1.4845E+04	8.4465E+03	6.9517E+03	1.4617E+04	1.4924E+04	1.0380E+04	1.3147E+04
	Std	4.8002E+02	9.0451E+02	3.7070E+02	8.1625E+02	4.8090E+02	9.1692E+02	8.7379E+02	6.1705E+02	3.7685E+02	1.5513E+03	6.2884E+02
	Rank	4	9	11	6	8	2	1	7	10	3	5
F10	Best	1.1579E+03	3.6201E+04	3.3295E+03	1.3852E+03	1.2554E+03	1.4220E+03	1.1898E+03	1.5687E+03	3.5517E+03	1.6061E+03	2.2059E+03
	Mean	1.2003E+03	5.5800E+04	4.1013E+03	1.5000E+03	1.3286E+03	1.7367E+03	1.2744E+03	1.7567E+03	5.0610E+03	1.8973E+03	2.9282E+03
	Std	2.4902E+01	6.7233E+03	6.8966E+02	7.0642E+01	3.1763E+01	1.2695E+02	4.1114E+01	1.3102E+02	9.2993E+02	2.5197E+02	4.8175E+02
	Rank	1	11	9	4	3	5	2	6	10	7	8
F11	Best	1.7189E+05	7.6264E+10	4.3218E+07	6.1703E+06	9.2833E+05	6.1401E+07	6.5903E+06	2.0549E+07	7.2555E+08	1.3711E+07	6.8476E+07
	Mean	5.5177E+05	1.0496E+11	6.7145E+07	1.6412E+07	2.5667E+06	1.9110E+08	2.7374E+07	5.0589E+07	1.4658E+09	4.7893E+07	1.6570E+08
	Std	3.4008E+05	1.5978E+10	1.3997E+07	9.6656E+06	1.0227E+06	1.1738E+08	2.0812E+07	2.3085E+07	4.6421E+08	2.8927E+07	6.1621E+07
	Rank	1	11	7	3	2	9	4	6	10	5	8
F12	Best	2.4391E+03	3.1843E+10	9.7256E+05	3.6086E+04	2.1042E+04	2.8426E+05	9.7950E+03	7.8852E+04	7.9005E+07	3.0591E+04	5.3090E+05
	Mean	3.5475E+03	4.9819E+10	1.6292E+06	1.0609E+05	3.6306E+04	9.6855E+05	4.5318E+04	3.4467E+05	1.9378E+08	9.4563E+04	1.8643E+06
	Std	7.0586E+02	1.2436E+10	4.0098E+05	5.0874E+04	8.3680E+03	5.2810E+05	3.2309E+04	2.3570E+05	6.5900E+07	4.0260E+04	1.1958E+06
	Rank	1	11	8	5	2	7	3	6	10	4	9
F13	Best	1.4751E+03	1.7495E+07	7.5992E+04	1.8250E+03	1.6287E+03	5.2149E+04	2.3804E+04	6.8934E+04	8.0445E+04	1.4352E+04	5.1818E+04
	Mean	1.5427E+03	1.3358E+08	2.6436E+05	3.4418E+03	1.6983E+03	3.4092E+05	2.5753E+05	2.5393E+05	3.6473E+05	1.9972E+05	2.0847E+05
	Std	3.6079E+01	7.6838E+07	9.4448E+04	2.5264E+03	3.3378E+01	2.2575E+05	1.7116E+05	2.0534E+05	1.6057E+05	2.0699E+05	1.3496E+05
	Rank	1	11	8	3	2	9	7	6	10	4	5
F14	Best	1.7004E+03	6.7712E+09	6.1112E+04	1.0822E+04	3.1595E+03	3.2584E+04	2.8235E+03	5.9753E+03	7.6169E+06	3.5969E+03	2.9355E+04
	Mean	1.8928E+03	1.8031E+10	1.5195E+05	3.2309E+04	4.9339E+03	2.4974E+05	1.8707E+04	1.3111E+04	2.7783E+07	1.2980E+04	7.3525E+04
	Std	1.1971E+02	5.3589E+09	6.0069E+04	1.2740E+04	1.0462E+03	5.0344E+05	1.9226E+04	4.9386E+03	1.5484E+07	9.1785E+03	3.9862E+04
	Rank	1	11	8	6	2	9	5	4	10	3	7
F15	Best	3.4129E+03	9.1935E+03	4.0010E+03	3.8105E+03	3.8013E+03	2.7224E+03	2.3367E+03	2.2976E+03	4.8427E+03	2.7697E+03	3.0889E+03
	Mean	4.1109E+03	1.1324E+04	4.4745E+03	4.4596E+03	4.5948E+03	3.6035E+03	3.3240E+03	3.1241E+03	5.2910E+03	3.5904E+03	3.5538E+03
	Std	2.3387E+02	1.2373E+03	2.5125E+02	3.0966E+02	2.8464E+02	4.1564E+02	4.2303E+02	4.7547E+02	2.2717E+02	4.8634E+02	3.3505E+02
	Rank	6	11	8	7	9	5	2	1	10	4	3
F16	Best	3.0677E+03	1.1176E+04	3.6479E+03	3.1624E+03	3.1645E+03	2.4416E+03	2.1071E+03	2.4097E+03	3.7035E+03	2.3757E+03	2.4259E+03
	Mean	3.4240E+03	1.1826E+05	3.8554E+03	3.5836E+03	3.7371E+03	3.1298E+03	2.9425E+03	2.9753E+03	4.1454E+03	3.0585E+03	3.1463E+03
	Std	1.6574E+02	6.0937E+04	1.1687E+02	2.3375E+02	2.0361E+02	3.3966E+02	3.0160E+02	3.6048E+02	1.6166E+02	3.2802E+02	2.7815E+02
	Rank	6	11	9	7	8	4	1	2	10	3	5
F17	Best	1.9983E+03	5.7610E+07	1.5850E+06	2.0840E+04	3.1707E+03	3.0820E+05	2.6616E+05	4.2225E+05	1.9810E+06	4.2380E+05	2.1801E+05
	Mean	2.1863E+03	3.6930E+08	2.6875E+06	5.7383E+04	5.8459E+03	4.1709E+06	3.1496E+06	2.1394E+06	5.7047E+06	2.0063E+06	2.1710E+06
	Std	1.5880E+02	2.0653E+08	8.1533E+05	2.2422E+04	1.8646E+03	3.0919E+06	2.0306E+06	2.0346E+06	2.7423E+06	1.5498E+06	1.1872E+06
	Rank	1	11	7	3	2	9	8	5	10	4	6
F18	Best	1.9594E+03	3.4516E+09	8.0256E+04	4.9139E+03	2.2565E+03	5.4001E+04	2.1806E+03	4.0608E+03	5.2122E+06	3.3964E+03	4.2786E+04
	Mean	2.0143E+03	7.6699E+09	1.8257E+05	2.5242E+04	2.9372E+03	1.5267E+06	1.4591E+04	1.6749E+04	1.2584E+07	1.4580E+04	3.6463E+05
	Std	4.1762E+01	1.8775E+09	5.3654E+04	1.4147E+04	8.3088E+02	1.6546E+06	1.2773E+04	8.4849E+03	6.2772E+06	1.0566E+04	3.6527E+05
	Rank	1	11	7	6	2	9	4	5	10	3	8
F19	Best	2.9200E+03	4.0413E+03	3.2258E+03	3.1455E+03	3.2645E+03	2.5615E+03	2.3627E+03	2.8742E+03	3.6984E+03	2.5155E+03	2.5675E+03
	Mean	3.4931E+03	4.7111E+03	3.9305E+03	3.6829E+03	3.9101E+03	3.1080E+03	2.9374E+03	3.6604E+03	4.0840E+03	3.1305E+03	3.2411E+03
	Std	1.7500E+02	3.0960E+02	2.2372E+02	2.9979E+02	1.9964E+02	2.5026E+02	3.3466E+02	3.3226E+02	1.8454E+02	3.2618E+02	3.0061E+02
	Rank	5	11	9	7	8	2	1	6	10	3	4
F20	Best	2.5322E+03	3.1403E+03	2.6315E+03	2.5413E+03	2.6125E+03	2.4545E+03	2.4365E+03	2.4674E+03	2.7067E+03	2.5095E+03	2.5361E+03
	Mean	2.5803E+03	3.3690E+03	2.6789E+03	2.6179E+03	2.6562E+03	2.5070E+03	2.4988E+03	2.5799E+03	2.7535E+03	2.5987E+03	2.5875E+03
	Std	1.5172E+01	8.0721E+01	1.5664E+01	3.3937E+01	1.7707E+01	3.8421E+01	3.7862E+01	5.0364E+01	2.1107E+01	4.0482E+01	2.6456E+01
	Rank	4	11	9	7	8	2	1	3	10	6	5
F21	Best	2.3001E+03	1.4843E+04	5.0061E+03	3.2616E+03	2.4737E+03	8.0262E+03	7.1865E+03	2.4908E+03	1.4945E+04	2.6251E+03	2.9254E+03
	Mean	1.2149E+04	1.7341E+04	1.5681E+04	1.5098E+04	1.5455E+04	1.0117E+04	8.8049E+03	7.5520E+03	1.6192E+04	1.1522E+04	1.3706E+04
	Std	3.2318E+03	9.2034E+02	2.5391E+03	2.3974E+03	3.5481E+03	9.4581E+02	7.9747E+02	5.7162E+03	4.7156E+02	3.2781E+03	3.0753E+03
	Rank	5	11	9	7	8	3	2	1	10	4	6
F22	Best	2.9380E+03	4.2234E+03	3.0593E+03	3.0025E+03	3.0430E+03	2.8524E+03	2.8584E+03	3.0462E+03	3.1503E+03	3.0080E+03	2.9719E+03
	Mean	3.0021E+03	4.7191E+03	3.1198E+03	3.0573E+03	3.0812E+03	2.9663E+03	2.9712E+03	3.1409E+03	3.2587E+03	3.1895E+03	3.0337E+03
	Std	2.1591E+01	2.3406E+02	2.6752E+01	2.7820E+01	2.0977E+01	4.8006E+01	5.3163E+01	5.7024E+01	3.6419E+01	7.5567E+01	3.8387E+01
	Rank	3	11	7	5	6	1	2	8	10	9	4
F23	Best	2.9416E+03	4.5226E+03	3.2146E+03	3.1332E+03	3.1888E+03	3.0049E+03	3.0283E+03	3.1904E+03	3.3291E+03	3.2322E+03	3.1274E+03
	Mean	3.1141E+03	5.1940E+03	3.2762E+03	3.2236E+03	3.2491E+03	3.1009E+03	3.1278E+03	3.3236E+03	3.4165E+03	3.3482E+03	3.1931E+03
	Std	6.4476E+01	2.5858E+02	2.3540E+01	3.7138E+01	1.7997E+01	4.4582E+01	5.5563E+01	6.6596E+01	3.5291E+01	8.7159E+01	3.0711E+01
	Rank	2	11	7	5	6	1	3	8	10	9	4
F24	Best	2.9736E+03	1.9442E+04	3.1934E+03	3.0671E+03	2.9916E+03	3.0419E+03	2.9379E+03	3.1365E+03	3.9980E+03	3.2247E+03	3.2646E+03
	Mean	3.0296E+03	1.9504E+04	3.2745E+03	3.0996E+03	3.0467E+03	3.1417E+03	3.0028E+03	3.2592E+03	4.6014E+03	3.3114E+03	3.4615E+03
	Std	2.1650E+01	9.1548E+01	3.7974E+01	1.6360E+01	2.1419E+01	5.4620E+01	4.6577E+01	5.3801E+01	3.6621E+02	5.9852E+01	9.7124E+01
	Rank	2	11	7	4	3	5	1	6	10	8	9
F25	Best	4.5423E+03	1.8852E+04	7.4764E+03	6.2607E+03	6.6373E+03	3.4882E+03	2.9006E+03	3.9090E+03	8.3870E+03	3.6995E+03	6.1016E+03
	Mean	6.0845E+03	1.9663E+04	7.8236E+03	6.7253E+03	7.0617E+03	6.0559E+03	4.1325E+03	7.1583E+03	9.1684E+03	7.5521E+03	6.8685E+03
	Std	4.0086E+02	2.7371E+02	1.9561E+02	2.7576E+02	1.8993E+02	6.0319E+02	1.8467E+03	1.5724E+03	3.4099E+02	2.4724E+03	4.3141E+02
	Rank	3	11	9	4	6	2	1	7	10	8	5
F26	Best	3.2448E+03	5.7410E+03	3.4828E+03	3.3078E+03	3.2444E+03	3.3422E+03	3.2000E+03	3.5779E+03	3.8527E+03	3.6345E+03	3.5373E+03
	Mean	3.3064E+03	7.4537E+03	3.6409E+03	3.4005E+03	3.2748E+03	3.4817E+03	3.2596E+03	3.7935E+03	4.0863E+03	3.8922E+03	3.6165E+03
	Std	4.2092E+01	5.8978E+02	8.2932E+01	4.9043E+01	2.5987E+01	8.0812E+01	5.6865E+01	1.0545E+02	1.2702E+02	1.6709E+02	5.8717E+01
	Rank	3	11	7	4	2	5	1	8	10	9	6
F27	Best	3.2593E+03	1.6734E+04	3.5239E+03	3.3414E+03	3.2650E+03	3.3573E+03	3.2563E+03	3.4409E+03	4.5866E+03	3.5337E+03	3.7823E+03
	Mean	3.2867E+03	1.9329E+04	3.6940E+03	3.3884E+03	3.3116E+03	3.4933E+03	3.2994E+03	3.6144E+03	5.3313E+03	3.7592E+03	4.0716E+03
	Std	2.3897E+01	1.1703E+03	1.0486E+02	2.3380E+01	2.1806E+01	8.1097E+01	3.0522E+01	1.1884E+02	4.0442E+02	1.4047E+02	1.8541E+02
	Rank	1	11	7	4	3	5	2	6	10	8	9
F28	Best	3.7557E+03	1.8325E+04	4.9786E+03	4.2669E+03	4.3046E+03	4.1416E+03	3.8382E+03	3.8890E+03	5.5302E+03	4.0426E+03	4.0729E+03
	Mean	4.1403E+03	2.9872E+05	5.4517E+03	4.8537E+03	4.8356E+03	4.7579E+03	4.3055E+03	4.5815E+03	6.2126E+03	4.8858E+03	4.5321E+03
	Std	2.2657E+02	2.6022E+05	2.3005E+02	2.5976E+02	2.1025E+02	3.9890E+02	3.3234E+02	3.6302E+02	3.1859E+02	4.8997E+02	3.2028E+02
	Rank	1	11	9	7	6	5	2	4	10	8	3
F29	Best	7.8257E+05	2.8747E+09	1.7100E+07	6.2304E+06	1.2985E+06	2.0167E+07	2.3065E+05	7.7012E+06	1.0520E+08	6.7975E+06	2.6337E+07
	Mean	9.8976E+05	1.1380E+10	2.5235E+07	9.5422E+06	1.9701E+06	5.3858E+07	3.1582E+06	1.6510E+07	1.7985E+08	1.7509E+07	5.0427E+07
	Std	1.3245E+05	3.3383E+09	4.4256E+06	2.5653E+06	3.3702E+05	2.3456E+07	1.9481E+06	4.2169E+06	4.5413E+07	7.6094E+06	1.7943E+07
	Rank	1	11	7	4	2	9	3	5	10	6	8

**Table A8:** The statistical results of MSDCS and competition algorithms for solving CEC2017 (D = 100).

Function	Index	MSDCS	DCS	AE	LSHADE	APSM-Jso	RIME	RDGMVO	QIO	MTVSCA	MRFO	EOSMA
F1	Best	1.3350E+04	2.9684E+11	2.6090E+09	2.9174E+08	8.0595E+06	8.5841E+08	3.0785E+05	2.2240E+09	3.8284E+10	7.6353E+09	1.5183E+10
	Mean	2.9247E+04	2.9764E+11	3.6879E+09	4.9785E+08	1.5859E+07	1.4656E+09	3.3567E+06	3.3316E+09	5.5903E+10	1.2933E+10	2.1570E+10
	Std	1.1532E+04	1.6287E+09	5.5874E+08	1.2670E+08	5.9913E+06	4.6316E+08	5.0088E+06	5.2953E+08	7.3869E+09	3.1514E+09	4.5282E+09
	Rank	1	11	7	4	3	5	2	6	10	8	9
F2	Best	4.7915E+04	3.6664E+05	2.9412E+05	1.4678E+05	4.6562E+04	2.5071E+05	1.4082E+05	2.8031E+05	2.7783E+05	2.5892E+05	2.9198E+05
	Mean	6.6450E+04	1.6169E+06	3.3820E+05	1.7255E+05	6.7962E+04	3.7506E+05	1.7517E+05	3.5681E+05	3.5498E+05	2.8973E+05	3.6448E+05
	Std	1.1398E+04	3.5697E+06	1.4640E+04	2.0569E+04	1.0281E+04	5.1070E+04	1.8732E+04	3.2608E+04	3.4888E+04	1.9080E+04	4.3300E+04
	Rank	1	11	6	3	2	10	4	8	7	5	9
F3	Best	6.2864E+02	1.2914E+05	1.1603E+03	7.8044E+02	6.9022E+02	9.2486E+02	5.5629E+02	1.1546E+03	4.8246E+03	1.9266E+03	2.0024E+03
	Mean	7.0445E+02	1.5964E+05	1.3172E+03	8.8262E+02	7.5663E+02	1.1549E+03	7.0548E+02	1.4575E+03	7.5043E+03	2.3761E+03	2.6752E+03
	Std	4.3913E+01	5.8754E+03	8.0855E+01	4.8852E+01	3.3427E+01	1.6176E+02	7.2881E+01	1.7453E+02	1.3289E+03	3.0475E+02	4.6118E+02
	Rank	1	11	6	4	3	5	2	7	10	8	9
F4	Best	1.1591E+03	2.3076E+03	1.3471E+03	1.1186E+03	1.2460E+03	9.3697E+02	9.3687E+02	1.1064E+03	1.5342E+03	1.2689E+03	1.1842E+03
	Mean	1.2082E+03	2.3276E+03	1.4489E+03	1.2015E+03	1.3060E+03	1.1018E+03	1.0623E+03	1.2590E+03	1.6018E+03	1.3688E+03	1.2910E+03
	Std	2.9011E+01	1.8753E+01	5.0899E+01	4.5381E+01	2.2262E+01	8.3525E+01	8.0549E+01	6.4184E+01	3.4505E+01	5.4262E+01	4.9968E+01
	Rank	4	11	9	3	7	2	1	5	10	8	6
F5	Best	6.0009E+02	7.2008E+02	6.1287E+02	6.0862E+02	6.0166E+02	6.2507E+02	6.1001E+02	6.3322E+02	6.4475E+02	6.5439E+02	6.2587E+02
	Mean	6.0019E+02	7.2459E+02	6.1626E+02	6.1154E+02	6.0259E+02	6.3379E+02	6.2418E+02	6.4223E+02	6.5645E+02	6.6531E+02	6.3553E+02
	Std	4.9376E-02	3.7810E+00	1.5777E+00	1.5940E+00	5.3474E-01	4.0863E+00	6.1948E+00	5.9726E+00	4.5676E+00	6.9985E+00	4.5964E+00
	Rank	1	11	4	3	2	6	5	8	9	10	7
F6	Best	1.4090E+03	4.2344E+03	1.6990E+03	1.4690E+03	1.5474E+03	1.6492E+03	1.2379E+03	1.7421E+03	2.2776E+03	2.1412E+03	1.7926E+03
	Mean	1.4928E+03	4.3151E+03	1.7786E+03	1.5668E+03	1.6237E+03	1.8791E+03	1.4988E+03	1.9292E+03	2.4752E+03	2.6372E+03	1.9948E+03
	Std	2.6487E+01	4.4668E+01	2.9735E+01	4.7091E+01	2.8969E+01	1.1242E+02	1.5958E+02	7.2153E+01	1.2859E+02	2.5664E+02	9.0620E+01
	Rank	1	11	5	3	4	6	2	7	9	10	8
F7	Best	1.4618E+03	2.7360E+03	1.6554E+03	1.4301E+03	1.5353E+03	1.2154E+03	1.1941E+03	1.4953E+03	1.8261E+03	1.5400E+03	1.4679E+03
	Mean	1.5050E+03	2.7783E+03	1.7483E+03	1.5189E+03	1.6056E+03	1.3972E+03	1.3404E+03	1.6019E+03	1.9144E+03	1.7316E+03	1.5797E+03
	Std	2.3491E+01	4.2030E+01	3.7612E+01	5.1068E+01	2.8396E+01	8.8138E+01	8.2532E+01	6.0702E+01	4.0603E+01	1.0080E+02	5.6604E+01
	Rank	3	11	9	4	7	2	1	6	10	8	5
F8	Best	9.3759E+02	1.0399E+05	5.4226E+03	1.9435E+03	9.4932E+02	1.3390E+04	1.2086E+04	1.2195E+04	2.8904E+04	3.1287E+04	1.2322E+04
	Mean	9.9044E+02	1.1248E+05	6.8974E+03	3.0474E+03	1.0389E+03	2.6553E+04	2.4118E+04	2.8892E+04	3.4249E+04	4.9525E+04	2.3193E+04
	Std	3.9379E+01	6.0070E+03	1.2710E+03	6.5567E+02	6.1779E+01	9.2911E+03	5.1026E+03	7.6037E+03	3.0445E+03	9.7716E+03	5.3367E+03
	Rank	1	11	4	3	2	7	6	8	9	10	5
F9	Best	2.4778E+04	2.9275E+04	2.9969E+04	2.5728E+04	3.0376E+04	1.6730E+04	1.1350E+04	2.9385E+04	3.0253E+04	1.6105E+04	2.5259E+04
	Mean	2.5680E+04	3.1473E+04	3.1819E+04	2.9168E+04	3.1842E+04	1.9530E+04	1.4314E+04	3.1258E+04	3.1654E+04	2.1769E+04	2.8185E+04
	Std	4.5864E+02	1.2612E+03	7.5126E+02	1.3505E+03	5.5021E+02	1.6465E+03	1.3525E+03	7.5523E+02	6.1466E+02	3.5483E+03	1.4281E+03
	Rank	4	8	10	6	11	2	1	7	9	3	5
F10	Best	1.9592E+03	2.4012E+05	4.9933E+04	4.0350E+03	2.1346E+03	7.1013E+03	2.2200E+03	3.7837E+04	4.0356E+04	5.2662E+04	3.8626E+04
	Mean	2.2330E+03	5.0035E+05	6.7846E+04	6.2837E+03	2.5840E+03	1.1354E+04	2.7605E+03	6.1852E+04	7.3882E+04	6.5593E+04	5.9875E+04
	Std	1.8827E+02	1.4673E+05	1.2338E+04	1.4875E+03	2.0304E+02	2.9310E+03	2.8835E+02	1.4458E+04	1.1044E+04	8.1461E+03	1.1507E+04
	Rank	1	11	9	4	2	5	3	7	10	8	6
F11	Best	1.2667E+06	2.0781E+11	4.8488E+08	3.9720E+07	8.8760E+06	3.6094E+08	2.8820E+07	3.0530E+08	6.5630E+09	3.9521E+08	8.9452E+08
	Mean	4.7159E+06	2.5449E+11	6.1856E+08	1.1104E+08	2.3810E+07	9.0860E+08	1.8844E+08	5.8245E+08	8.8107E+09	7.2175E+08	1.3499E+09
	Std	2.5222E+06	1.4336E+10	8.7346E+07	4.2713E+07	1.0507E+07	3.3934E+08	8.4431E+07	1.8915E+08	1.7059E+09	2.0701E+08	2.9808E+08
	Rank	1	11	6	3	2	8	4	5	10	7	9
F12	Best	3.5128E+03	3.5757E+10	1.4217E+06	3.2577E+04	2.3581E+04	8.5149E+05	2.0374E+04	5.9674E+05	2.0921E+08	1.9798E+05	2.5229E+06
	Mean	7.5280E+03	6.2823E+10	2.6871E+06	4.7951E+04	3.1703E+04	3.1786E+06	5.2340E+04	1.1260E+06	5.8124E+08	5.8425E+05	7.9870E+06
	Std	2.9964E+03	5.7740E+09	6.1405E+05	9.9427E+03	5.8564E+03	1.8120E+06	2.5284E+04	2.9597E+05	2.2985E+08	2.4476E+05	3.8984E+06
	Rank	1	11	7	3	2	8	4	6	10	5	9
F13	Best	1.6734E+03	1.6766E+08	1.6814E+06	8.9207E+03	2.0717E+03	1.1704E+06	3.3716E+05	9.0726E+05	3.0731E+06	7.3809E+05	1.4504E+06
	Mean	1.8192E+03	3.0448E+08	2.8006E+06	4.0016E+04	2.2824E+03	4.6634E+06	1.6296E+06	1.6692E+06	4.8133E+06	2.0851E+06	3.6329E+06
	Std	9.2321E+01	3.4149E+07	6.3487E+05	2.3752E+04	1.5681E+02	2.1901E+06	9.0448E+05	5.8006E+05	1.1471E+06	1.0199E+06	1.3470E+06
	Rank	1	11	7	3	2	9	4	5	10	6	8
F14	Best	2.0984E+03	2.3488E+10	1.7170E+05	1.8415E+04	9.8597E+03	1.3014E+05	1.3160E+04	2.3144E+04	2.8296E+07	1.8102E+04	1.2471E+05
	Mean	2.5738E+03	3.4015E+10	2.3135E+05	2.8697E+04	1.7787E+04	5.8427E+05	3.3717E+04	5.4552E+04	1.0111E+08	3.1364E+04	2.7507E+05
	Std	4.7907E+02	4.7886E+09	3.5246E+04	7.9118E+03	3.7618E+03	4.0695E+05	1.4200E+04	1.5301E+04	5.2012E+07	1.0669E+04	1.2804E+05
	Rank	1	11	7	3	2	9	5	6	10	4	8
F15	Best	7.1139E+03	2.1235E+04	8.7712E+03	6.4232E+03	8.3254E+03	4.6307E+03	3.8843E+03	4.6268E+03	9.1373E+03	4.5869E+03	5.2187E+03
	Mean	8.3202E+03	3.0307E+04	9.5862E+03	8.3067E+03	9.1585E+03	6.5446E+03	5.4145E+03	5.9189E+03	1.0795E+04	6.4693E+03	7.0781E+03
	Std	5.0722E+02	3.7320E+03	3.4883E+02	6.7186E+02	3.8193E+02	8.9291E+02	7.7728E+02	7.4864E+02	5.1507E+02	9.0729E+02	6.8898E+02
	Rank	7	11	9	6	8	4	1	2	10	3	5
F16	Best	5.7313E+03	3.0078E+06	5.9553E+03	5.4573E+03	6.1223E+03	4.2969E+03	3.8511E+03	4.0112E+03	7.2091E+03	3.7855E+03	3.5796E+03
	Mean	6.3100E+03	1.6464E+07	6.8110E+03	6.3693E+03	6.7019E+03	5.3021E+03	4.8435E+03	4.9971E+03	7.6751E+03	5.0748E+03	5.0650E+03
	Std	2.8006E+02	1.2849E+07	2.8416E+02	2.9334E+02	2.6908E+02	4.3439E+02	6.1655E+02	7.3385E+02	2.7347E+02	6.2899E+02	5.4916E+02
	Rank	6	11	9	7	8	5	1	2	10	4	3
F17	Best	5.5823E+03	2.4359E+08	1.4635E+06	8.1939E+04	1.7603E+04	2.4134E+06	1.0154E+06	1.7058E+06	3.8746E+06	1.0686E+06	2.0002E+06
	Mean	1.5792E+04	6.2649E+08	2.9510E+06	1.5400E+05	3.6271E+04	6.9883E+06	2.7154E+06	3.1938E+06	7.4320E+06	3.0362E+06	4.0813E+06
	Std	6.7405E+03	2.0734E+08	6.1231E+05	5.4708E+04	1.1151E+04	3.8996E+06	1.4284E+06	9.9958E+05	3.2136E+06	1.5342E+06	1.7649E+06
	Rank	1	11	5	3	2	9	4	7	10	6	8
F18	Best	2.1620E+03	2.1978E+10	4.9815E+05	3.6823E+04	5.9842E+03	1.7404E+06	6.0233E+03	5.0827E+04	3.1849E+07	1.8310E+04	7.5135E+05
	Mean	2.5742E+03	3.4442E+10	1.1954E+06	1.1958E+05	1.0889E+04	9.8199E+06	1.5620E+05	1.1335E+05	1.1486E+08	4.6588E+04	2.5222E+06
	Std	5.8195E+02	5.3839E+09	2.5174E+05	5.0477E+04	3.2431E+03	5.2220E+06	1.6908E+05	4.7776E+04	5.4582E+07	1.9119E+04	1.5209E+06
	Rank	1	11	7	5	2	9	6	4	10	3	8
F19	Best	5.4619E+03	7.4799E+03	5.9137E+03	5.6594E+03	6.4142E+03	3.6402E+03	4.0073E+03	5.0120E+03	7.1349E+03	4.1843E+03	5.0990E+03
	Mean	6.6378E+03	8.4735E+03	7.2558E+03	6.5595E+03	7.3112E+03	5.1300E+03	5.0291E+03	7.0399E+03	7.5837E+03	5.2535E+03	5.9544E+03
	Std	3.3192E+02	4.2787E+02	3.4993E+02	5.9728E+02	2.6479E+02	5.7097E+02	4.6037E+02	5.2714E+02	2.2483E+02	5.0979E+02	4.2577E+02
	Rank	6	11	8	5	9	2	1	7	10	3	4
F20	Best	2.9730E+03	4.6856E+03	3.0790E+03	2.9175E+03	3.0559E+03	2.7736E+03	2.6847E+03	2.9463E+03	3.3409E+03	3.0479E+03	2.9842E+03
	Mean	3.0369E+03	5.0808E+03	3.2159E+03	3.0310E+03	3.1277E+03	2.9441E+03	2.8131E+03	3.1630E+03	3.4260E+03	3.2314E+03	3.0832E+03
	Std	2.7201E+01	1.6133E+02	4.8394E+01	4.4675E+01	2.6831E+01	6.4723E+01	7.8292E+01	1.0070E+02	4.0333E+01	9.7268E+01	5.6106E+01
	Rank	4	11	8	3	6	2	1	7	10	9	5
F21	Best	2.6611E+04	3.1184E+04	3.2979E+04	2.8966E+04	3.2107E+04	1.8578E+04	1.3726E+04	4.3499E+03	3.3217E+04	2.0156E+04	2.9348E+04
	Mean	2.8066E+04	3.3962E+04	3.4045E+04	3.2062E+04	3.4017E+04	2.1706E+04	1.6985E+04	2.9588E+04	3.4046E+04	2.3821E+04	3.1126E+04
	Std	6.5354E+02	1.6898E+03	4.2626E+02	1.4972E+03	5.7174E+02	1.4694E+03	1.6378E+03	9.6311E+03	4.1340E+02	2.4045E+03	1.0010E+03
	Rank	4	8	10	7	9	2	1	5	11	3	6
F22	Best	3.2917E+03	6.3739E+03	3.6917E+03	3.4780E+03	3.5852E+03	3.2571E+03	3.1788E+03	3.8314E+03	3.9714E+03	3.8453E+03	3.5059E+03
	Mean	3.4768E+03	7.2035E+03	3.7715E+03	3.5989E+03	3.6495E+03	3.4058E+03	3.2992E+03	4.0536E+03	4.0920E+03	4.0494E+03	3.5918E+03
	Std	6.3651E+01	3.6756E+02	4.0248E+01	5.4635E+01	2.6604E+01	7.6410E+01	9.0792E+01	9.0025E+01	4.8070E+01	1.3431E+02	6.5157E+01
	Rank	3	11	7	5	6	2	1	9	10	8	4
F23	Best	3.4257E+03	1.0259E+04	4.0767E+03	3.8864E+03	3.8809E+03	3.7490E+03	3.7149E+03	4.4364E+03	4.6004E+03	4.4583E+03	3.8561E+03
	Mean	3.8404E+03	1.2160E+04	4.1710E+03	4.0282E+03	4.0436E+03	3.9611E+03	3.8646E+03	4.7078E+03	4.7680E+03	4.9988E+03	4.1086E+03
	Std	1.7943E+02	7.5770E+02	5.4600E+01	5.8159E+01	4.7968E+01	1.0300E+02	1.0616E+02	1.5237E+02	8.0013E+01	2.3621E+02	8.5560E+01
	Rank	1	11	7	4	5	3	2	8	9	10	6
F24	Best	3.2107E+03	3.5072E+04	4.0130E+03	3.4843E+03	3.3492E+03	3.6022E+03	3.2535E+03	3.8445E+03	6.7517E+03	4.2522E+03	4.5991E+03
	Mean	3.3364E+03	3.5190E+04	4.1584E+03	3.5901E+03	3.4472E+03	3.9110E+03	3.3324E+03	4.1443E+03	8.5113E+03	4.8992E+03	5.2514E+03
	Std	4.4704E+01	2.8393E+02	8.0259E+01	4.5029E+01	3.5417E+01	1.3813E+02	4.5147E+01	1.5060E+02	7.9161E+02	3.6275E+02	3.0451E+02
	Rank	2	11	7	4	3	5	1	6	10	8	9
F25	Best	7.5090E+03	5.8927E+04	1.3554E+04	1.1051E+04	1.2033E+04	1.1329E+04	2.9093E+03	1.4576E+04	1.8088E+04	1.7904E+04	1.3352E+04
	Mean	1.0466E+04	5.9128E+04	1.4982E+04	1.2740E+04	1.3078E+04	1.2961E+04	1.1075E+04	1.7094E+04	1.9747E+04	2.2545E+04	1.5816E+04
	Std	1.5928E+03	2.8464E+02	6.1984E+02	6.8543E+02	4.5939E+02	9.8637E+02	2.9500E+03	1.4330E+03	9.1925E+02	2.1674E+03	1.4620E+03
	Rank	1	11	6	3	5	4	2	8	9	10	7
F26	Best	3.4145E+03	1.1040E+04	3.5954E+03	3.4616E+03	3.3259E+03	3.5830E+03	3.2000E+03	3.8978E+03	4.6568E+03	4.0930E+03	3.7876E+03
	Mean	3.5102E+03	1.4078E+04	3.6787E+03	3.5132E+03	3.3865E+03	3.8099E+03	3.4104E+03	4.1924E+03	5.0614E+03	4.4765E+03	3.9900E+03
	Std	4.5557E+01	1.0569E+03	4.9431E+01	4.2008E+01	3.5469E+01	1.1238E+02	9.9408E+01	1.5809E+02	2.0915E+02	2.3876E+02	7.8490E+01
	Rank	3	11	5	4	1	6	2	8	10	9	7
F27	Best	3.4018E+03	3.3561E+04	4.2417E+03	3.6196E+03	3.4565E+03	3.8013E+03	3.3000E+03	4.0923E+03	9.4709E+03	4.8327E+03	5.6504E+03
	Mean	3.4740E+03	3.3676E+04	4.7669E+03	3.7329E+03	3.5146E+03	4.3922E+03	3.3829E+03	4.4410E+03	1.1528E+04	6.0752E+03	6.9347E+03
	Std	3.7747E+01	1.7309E+02	1.9307E+02	7.8632E+01	3.6432E+01	3.3406E+02	4.8152E+01	2.9596E+02	1.2756E+03	7.0412E+02	7.0042E+02
	Rank	2	11	7	4	3	5	1	6	10	8	9
F28	Best	6.4399E+03	5.1267E+05	9.1981E+03	7.4407E+03	7.7059E+03	6.8929E+03	5.2296E+03	6.9089E+03	1.0621E+04	6.7636E+03	6.7368E+03
	Mean	7.6775E+03	2.5900E+06	9.6933E+03	8.3053E+03	8.5328E+03	8.0339E+03	6.6144E+03	7.9841E+03	1.1761E+04	8.4543E+03	7.8698E+03
	Std	5.6542E+02	1.5417E+06	3.1795E+02	5.0628E+02	3.2060E+02	5.2575E+02	5.8245E+02	6.1511E+02	4.7623E+02	6.3440E+02	6.5880E+02
	Rank	2	11	9	6	8	5	1	4	10	7	3
F29	Best	1.6410E+04	3.1111E+10	1.3787E+07	8.3148E+05	1.5889E+05	4.9164E+07	8.3244E+05	4.0824E+06	2.1821E+08	2.6460E+06	1.5559E+07
	Mean	2.4630E+04	5.3962E+10	1.8034E+07	1.7651E+06	2.7604E+05	1.2455E+08	3.0859E+06	7.5934E+06	3.7668E+08	6.1551E+06	3.9944E+07
	Std	5.7492E+03	7.5814E+09	3.0011E+06	6.4832E+05	6.9520E+04	5.1296E+07	2.0484E+06	2.6471E+06	1.2285E+08	2.9525E+06	1.6127E+07
	Rank	1	11	7	3	2	9	4	6	10	5	8
